# Supplementary material for: Tuning porosity in macroscopic monolithic metal-organic frameworks for exceptional natural gas storage
Source: Nat Commun. 2019 May 28;10:2345. doi: 10.1038/s41467-019-10185-1 (PMC6538620; doi:10.1038/s41467-019-10185-1)
Supplement: Supplementary file 1 — Supplementary Information [file 41467_2019_10185_MOESM1_ESM.pdf]

Supplementary Information

Tuning porosity in macroscopic monolithic metal-organic frameworks for exceptional natural gas storage

Connolly et al.

Supplementary Table 1| Experimental conditions for $_{mono}$UiO-66 synthesis. UiO-66 gel was synthesised from a modified literature procedure[1], and washed and dried under a range of synthetic conditions.

	Washing procedure	Centrifugation procedure	Drying temperature (°C)
UiO-66_A	Ethanol (3 × 30 ml)	3 × 10 min*	200
UiO-66_B	Ethanol (3 × 30 ml)	3 × 10 min*	30
UiO-66_C	DMF (1 × 30 ml)	1 × 10 min*	30
UiO-66_D	DMF (1 × 30 ml)	1 x 10 min* + 1 x 180 min**	30

*Centrifugation (5500 rpm) performed after each wash to re-obtain MOF gel as sediment.

**Additional 180 min (5500 rpm) centrifugation performed on densified MOF gel after washing in DMF and decanting the supernatant.

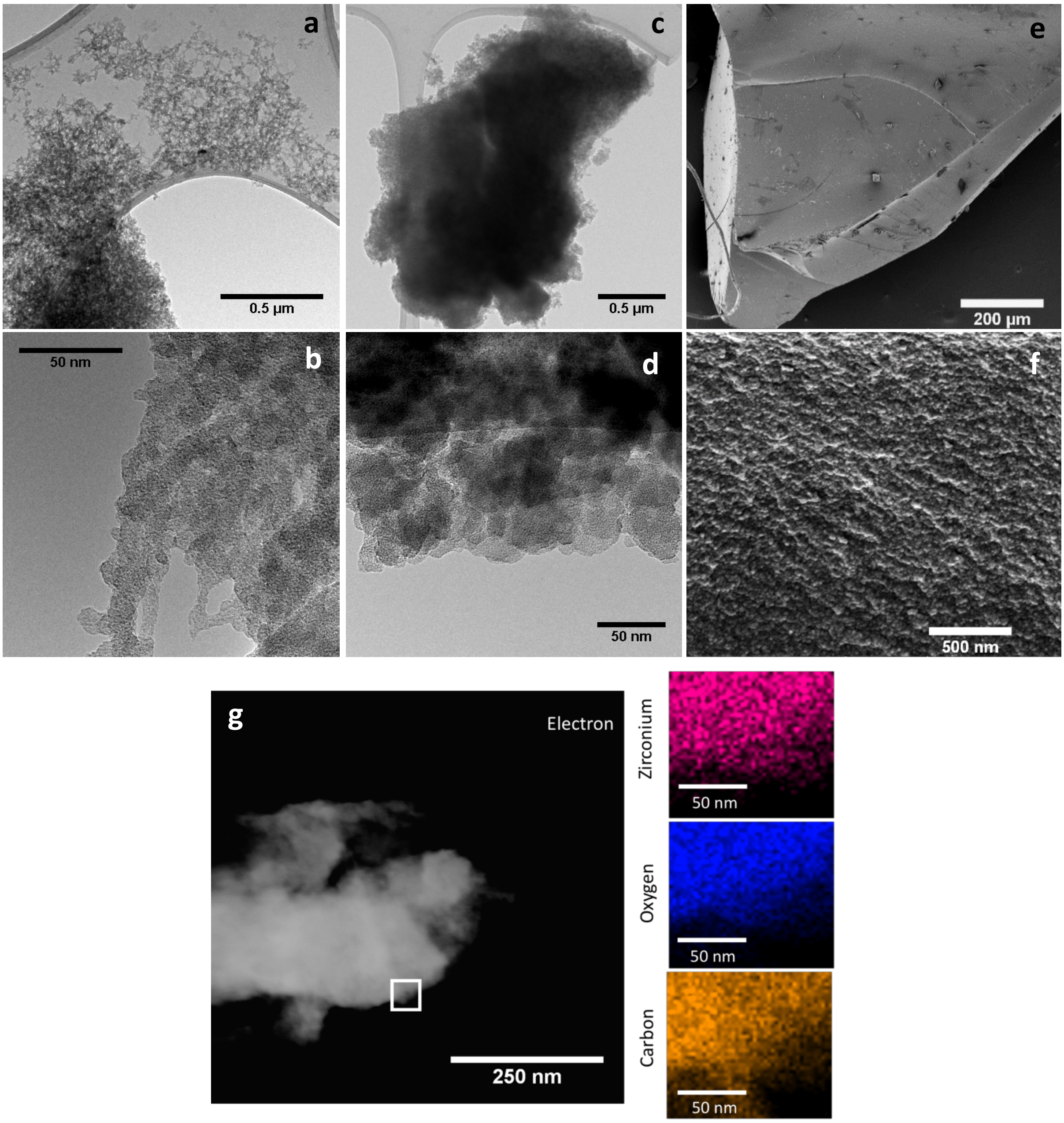

Supplementary Figure 1| $_{mono}$UiO-66 electron microscopy. a, b, TEM images of UiO-66 gel at low and high magnification, respectively. A sample of MOF gel was diluted in ethanol and placed on a TEM grid. The irregularly shaped MOF primary nanoparticles adopt a gelatinous network macrostructure. This prevents high accuracy size measurements. **c, d,** TEM images of fully dried densified monolith (UiO-66_D) at low and high magnification, respectively. A sample of the material was crushed with a spatula and a TEM grid was gently pressed into the resulting powder. **e,** A SEM image of a dried monolith and **f, a** high magnification SEM image showing surface texture comprised of densely packed primary particles. A monolith sample was stuck to a SEM stub using conductive carbon tape. **g,** A low magnification STEM electron image of a monolith (UiO-66_D) highlighting the area selected for EDX analysis (white box) and the corresponding EDX elemental maps showing the distribution of zirconium (pink), oxygen (blue) and carbon (orange).

Supplementary Table 2| Elemental analysis of $_{mono}$UiO-66. The experimentally determined elemental composition (%) of $_{mono}$UiO-66 was obtained by inductively coupled plasma-optical emission spectroscopy (ICP-OES, see 'Methods'). The theoretical composition was calculated from the chemical formula ($Zr_6(\mu_3$-$OH)_4(\mu_3$-$O)_4(BDC)_6$, H_2BDC = 1,4-benzenedicarboxylic acid). All experimentally obtained samples matched satisfactorily with the theoretical elemental composition and no significant compositional differences were observed for the different samples (UiO-66_A-D). Experimental deviation from the theoretical elemental composition (calculated from a perfect crystal structure) can result from defects in the crystal structure. In this case, missing linker defects are suggested by the lower experimentally observed carbon composition relative to the theoretical composition. This defect is commonly reported for UiO-66.[2] Additionally, the increased hydrogen content of the experimental samples may indicate the presence of water in the crystal structure. Although all samples were thoroughly degassed prior to analysis, foreign species from the air can be adsorbed by the MOFs during the ICP-OES sample preparation procedure (weighing under ambient conditions).

	Composition (%)				
	Theoretical	UiO-66_A	UiO-66_B	UiO-66_C	UiO-66_D
Zirconium	32.9	31.2	29.7	31.6	31.7
Carbon	34.6	31.9	31.7	32.0	32.0
Hydrogen	1.7	2.1	2.2	2.0	2.1
Nitrogen	0.0	0.0	0.0	0.3	0.0

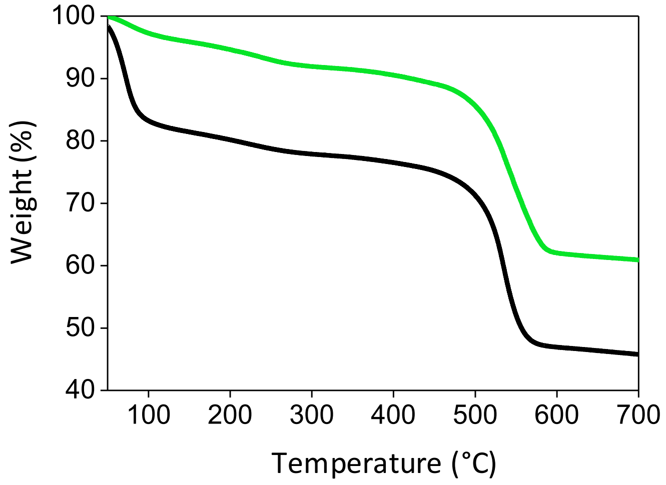

Supplementary Figure 2| Thermogravimetric analysis of $_{mono}$UiO-66. Thermogravimetric analysis (TGA) comparison (50-700 °C) of $_{mono}$UiO-66_D (green) with a powdered UiO-66 sample (black) prepared according to a procedure reported in literature.[3] The decomposition temperature (ca. 550 °C) is unchanged between samples, showing there to be no alteration to the thermal stability as a result of monolith formation. The initial weight loss (%) observed in the powdered sample at temperatures < 100 °C can be attributed to a loss of foreign species (e.g. water) adsorbed by the non-densified, porous sample from the air before the measurement was taken. The greater external surface area of the powdered material may facilitate faster uptake of foreign species into pores than in the corresponding monolith of the same material, which displays reduced adsorption kinetics.

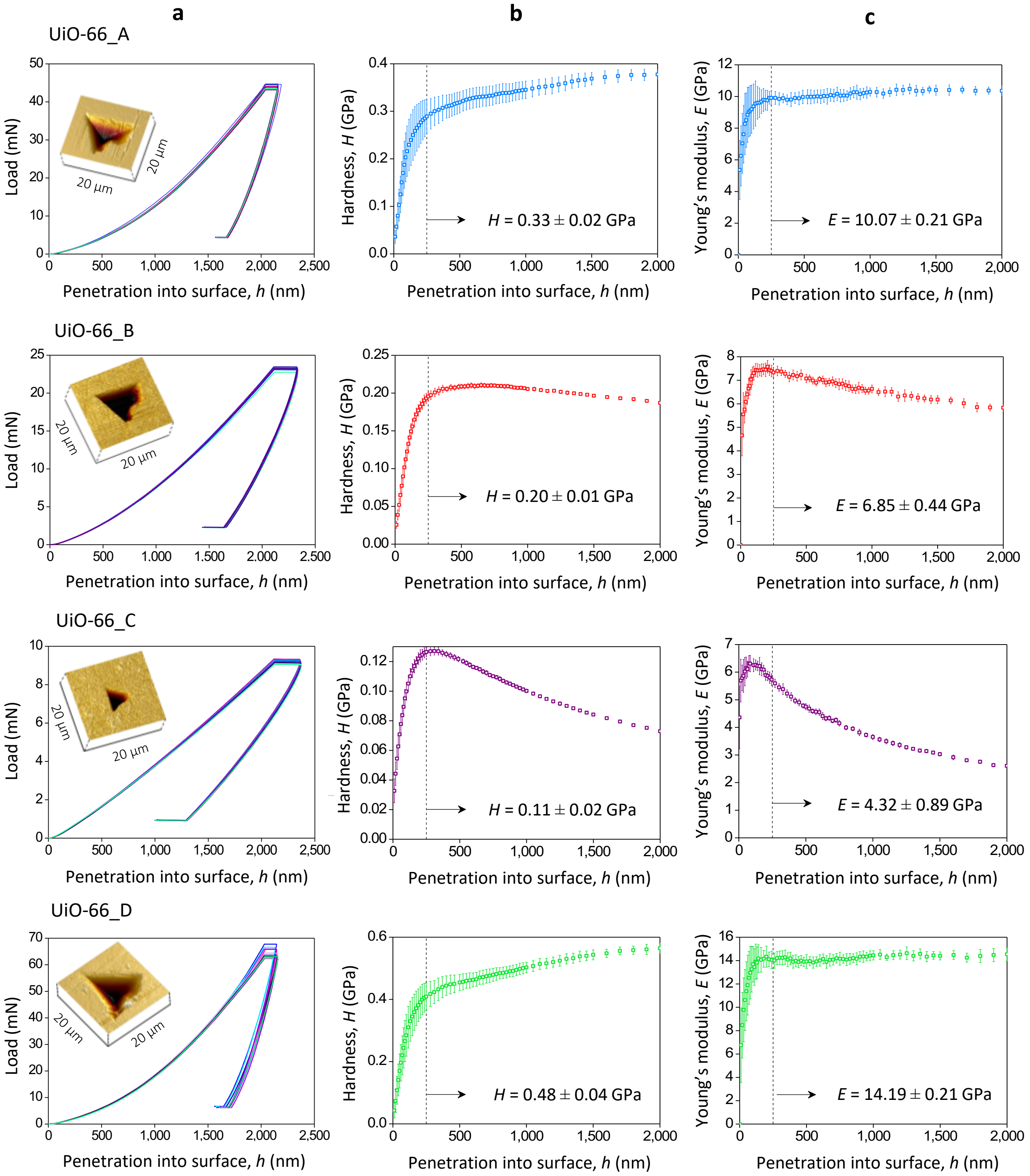

Supplementary Figure 3| Mechanical testing of $_{mono}$UiO-66. Mechanical testing of $_{mono}$UiO-66_A-D was performed via nanoindentation (see 'Methods') **a,** A plot of the Load (mN) vs. Penetration into surface of the monolith (*h*, nm) is shown for 16 independent indents. The insets in **a,** are a 3D rendering of the atomic force microscopy (AFM) images, showing the 3D topography of a resulting surface indent. The absence of radial cracking about the point of contact suggests good mechanical stability in each of the materials. Streaks in the image are an imaging artefact arising from the 'dragging' of unavoidable contaminants (resulting from the mechanical and chemical polishing sample preparation procedure, see 'Methods') over the surface by the microscope tip. **b, c,** The Hardness (*H*, GPa) and Young's modulus (*E*, GPa), respectively, are plotted as a function of Penetration depth (nm) into the monolith surface. The mean properties and corresponding errors (inset in **b** and **c**) were obtained via measurements taken from 16 indents over penetration depths of 250-2000 nm. Measurements obtained in the sub 250 nm penetration range were excluded to eliminate errors due to surface defects/tip artefacts. Despite UiO-66 traditionally exhibiting high mechanical strength (attributable to the presence of 12 hard-hard Zr-O coordination bonds per metal cluster),[4] literature mechanical properties vary

significantly.[5] For example, missing-linker defects are reported to reduce mechanical stability of MOFs whereas the presence of a residual modulator within the MOF structure has been reported to increase its stability.[6] Significantly, the mechanical properties of all samples reported here are comparable to those of robust monoliths we have previously reported: $_{mono}$ZIF-8 (E = 3.57 ± 0.22 GPa, H = 0.429 ± 0.026 GPa)[7] and $_{mono}$HKUST-1 (E = 9.3 ± 0.3 GPa, H = 0.46 ± 0.03 GPa).[8]

Supplementary Notes 1| Fluorescence lifetime imaging microscopy. The FLIM phasor approach, as described by Digman and co-workers, was used for analyzing FLIM measurements.[9] This approach allows a graphical interpretation of the measured fluorescence lifetime, thus avoiding a more complicated fit-based analysis that can result in biased interpretation of the data when a wrong fit model is used. From the FLIM measurements, the sine and cosine Fourier components of the lifetime decay are calculated for every pixel of an image, yielding the two phasor coordinates *g* and *s*, calculated using Supplementary equations [1] and [2]:

$$g_{\mathrm{i,j}}(\omega) = \int_0^{2\pi} I_{\mathrm{i,j}}(t) \cdot \cos(\omega t - \varphi_{\mathrm{Inst}})\,\mathrm{d}t / \left(M_{\mathrm{Inst}} \cdot \int_0^{2\pi} I_{\mathrm{i,j}}(t)\mathrm{d}t\right) \quad \text{[Supplementary equation 1]}$$

$$s_{\mathrm{i,j}}(\omega) = \int_0^{2\pi} I_{\mathrm{i,j}}(t) \cdot \sin(\omega t - \varphi_{\mathrm{Inst}})\,\mathrm{d}t / \left(M_{\mathrm{Inst}} \cdot \int_0^{2\pi} I_{\mathrm{i,j}}(t)\mathrm{d}t\right) \quad \text{[Supplementary equation 2]}$$

where the indices i and j define the pixel of the image and $I_{\mathrm{i,j}}(t)$ gives the photon counts of the time bin, t, of the lifetime decay histogram of the corresponding pixel. The frequency, ω, corresponds to $2\pi/T$, with T being the full timescale of the lifetime decay histogram (here 40 ns). φ_{Inst} and M_{Inst} are correction terms for the phase shift and demodulation caused by the instrument response function. They were calculated using a reference sample with known lifetime (Atto 425, 3.6 ns, Atto-Tec).

From the two phasor coordinates, two lifetime values can be determined, one based on the phase (τ_{φ}) [Supplementary equation 3] and the other on the modulation (τ_{M}) [Supplementary equation 4]:

$$\tau_{\varphi}(\omega) = \frac{1}{\omega} \cdot \frac{s}{g} \quad \text{[Supplementary equation 3]}$$

$$\tau_{\mathrm{M}}(\omega) = \frac{1}{\omega}\sqrt{\frac{1}{g^2+s^2} - 1} \quad \text{[Supplementary equation 4]}$$

For a purely mono-exponential decay, these two lifetimes are identical and correspond to the correct lifetime. In the case of multi-exponential components, the phase and modulation lifetimes are different and do not correspond directly and unambiguously to the pure species. In the phasor approach, multiple species will be added vectorially. For example, a mixture of two species with different mono-exponential lifetimes will lie on a line connecting the individual components of the two species on the unit circle. Where the mixture falls on this line depends on the relative population of the two species. To get a single apparent lifetime for each sample, the mean τ_{φ} and τ_{M} were calculated from all pixels above a threshold of ~ 300 photons. The arithmetic average of the mean phase and modulation lifetimes was then used to calculate an apparent lifetime. The uncertainty corresponds to the standard deviation of the pixel distribution.

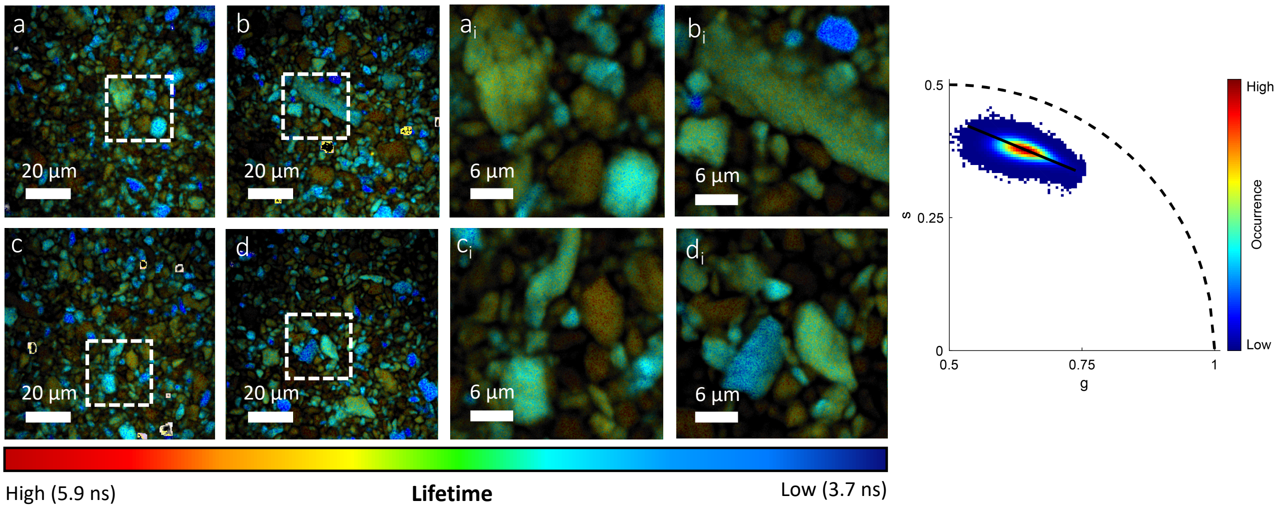

Supplementary Figure 4| FLIM studies of $_{mono}$UiO-66_A. (left) FLIM images (**a – d**), of $_{mono}$UiO-66_A showing the aggregated MOF primary particles which comprise the monolith. White dashed boxes indicate the area selected for high magnification imaging (**a_i – d_i**). The colours correspond to the fluorescence lifetime (see colour bar, below). (right) A 2D histogram phasor plot generated from the averaged FLIM images. The colours correspond to the frequency of occurrences (see colour bar, right).

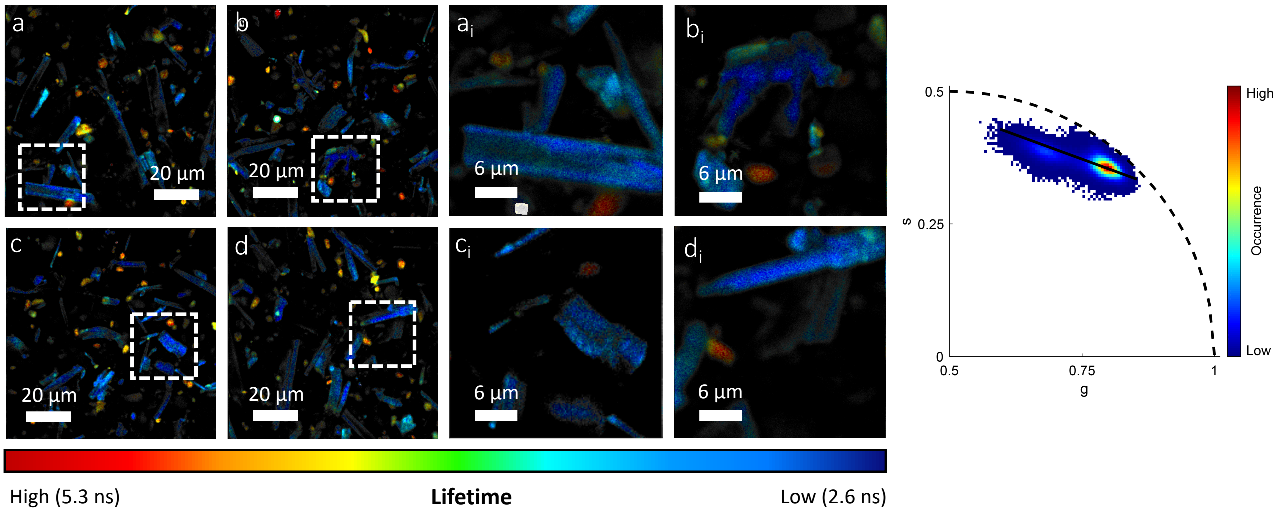

Supplementary Figure 5| FLIM studies of $_{mono}$UiO-66_B. (left) FLIM images (**a – d**), of $_{mono}$UiO-66_B showing the aggregated MOF primary particles which comprise the monolith. White dashed boxes indicate the area selected for high magnification imaging (**a_i – d_i**). The colours correspond to the fluorescence lifetime (see colour bar, below). (right) A 2D histogram phasor plot generated from the averaged FLIM images. The colours correspond to the frequency of occurrences (see colour bar, right).

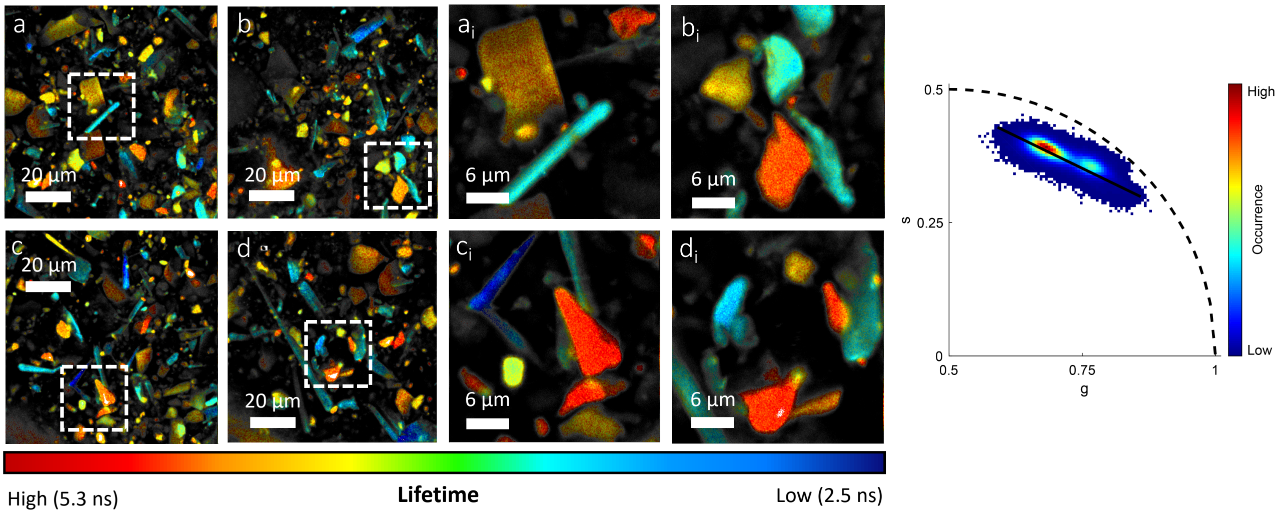

Supplementary Figure 6| FLIM studies of $_{mono}$UiO-66_C. (left) FLIM images (**a – d**), of $_{mono}$UiO-66_C showing the aggregated MOF primary particles which comprise the monolith. White dashed boxes indicate the area selected for high magnification imaging (**a$_i$ – d$_i$**). The colours correspond to the fluorescence lifetime (see colour bar, below). (right) A 2D histogram phasor plot generated from the averaged FLIM images. The colours correspond to the frequency of occurrences (see colour bar, right).

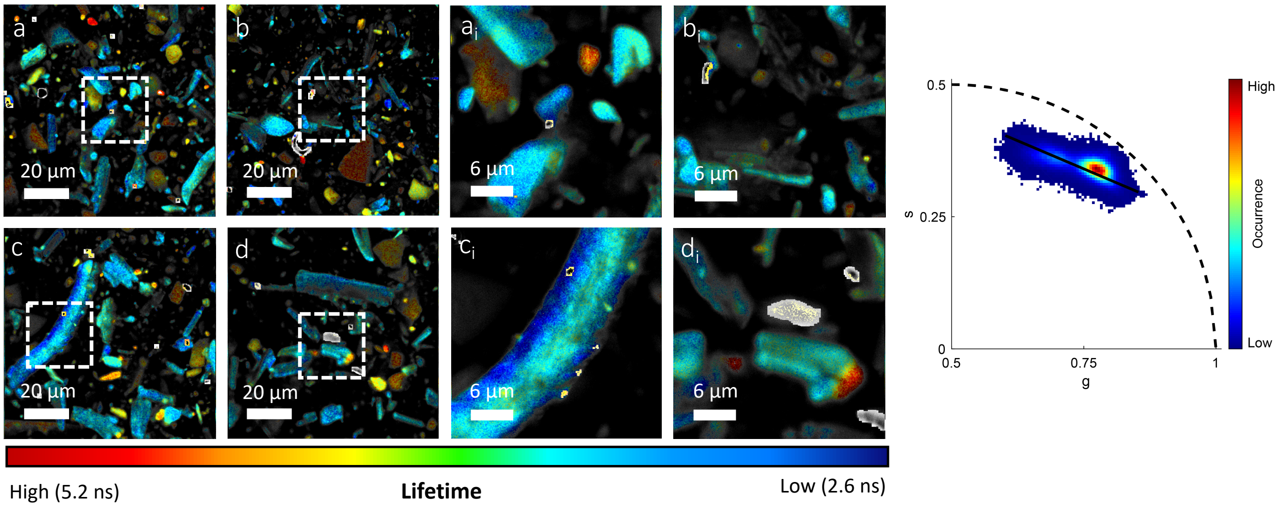

Supplementary Figure 7| FLIM studies of $_{mono}$UiO-66_D. (left) FLIM images (**a – d**), of $_{mono}$UiO-66_D showing the aggregated MOF primary particles which comprise the monolith. White dashed boxes indicate the area selected for high magnification imaging (**a$_i$ – d$_i$**). The colours correspond to the fluorescence lifetime (see colour bar, below). (right) A 2D histogram phasor plot generated from the averaged FLIM images. The colours correspond to the frequency of occurrences (see colour bar, right).

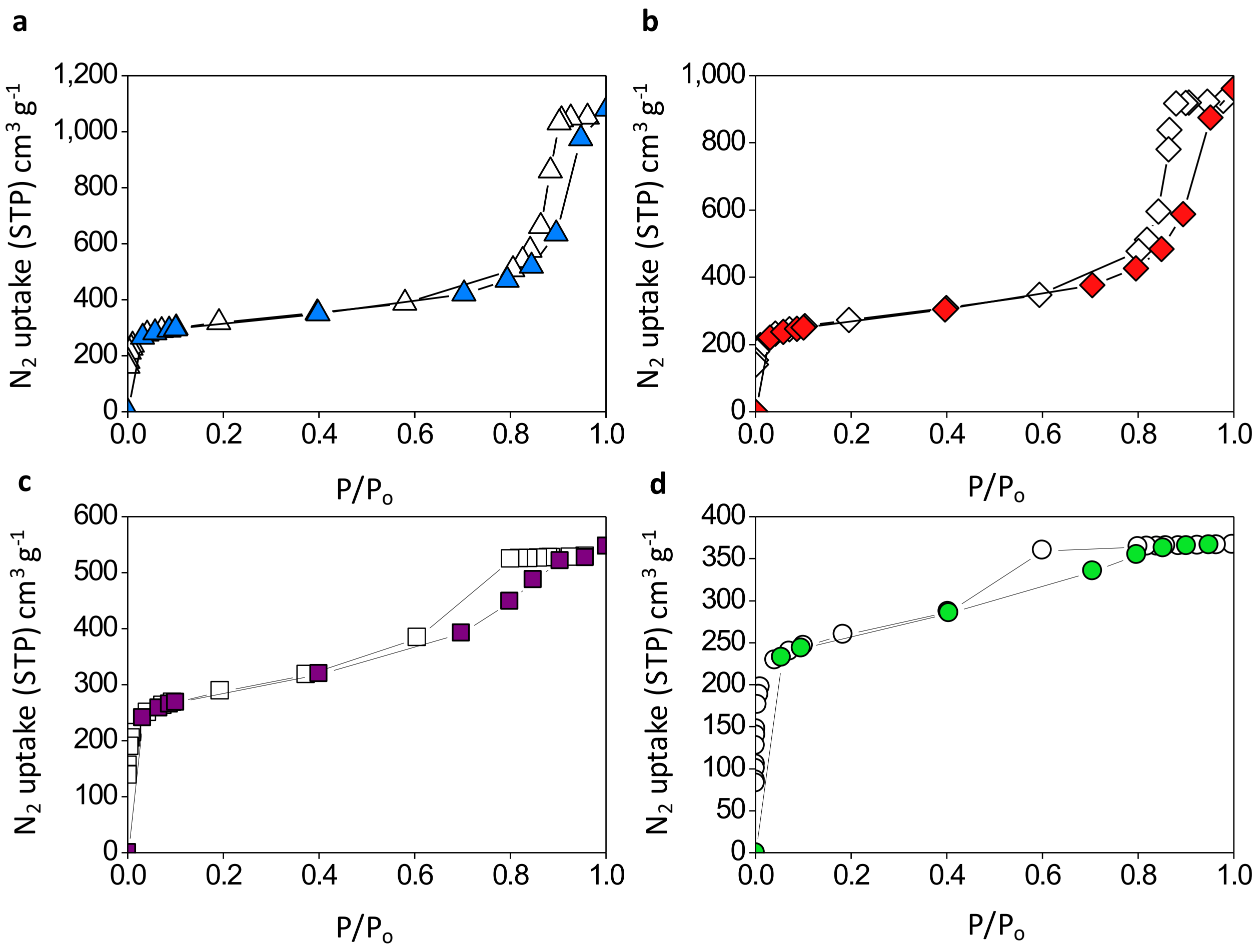

Supplementary Figure 8| N_2 desorption isotherms for $_{mono}$UiO-66. Adsorption (coloured marker) and desorption (white marker) N_2 isotherms collected for $_{mono}$UiO-66_A-D (0-1 bar at 77 K): **a,** UiO-66_A (blue); **b,** UiO-66_B (red); **c,** UiO-66_C (purple) and **d,** UiO-66_D (green). Hysteretic gas uptake/release was observed in all monoliths in both the micro- and mesoporous associated pressure ranges.

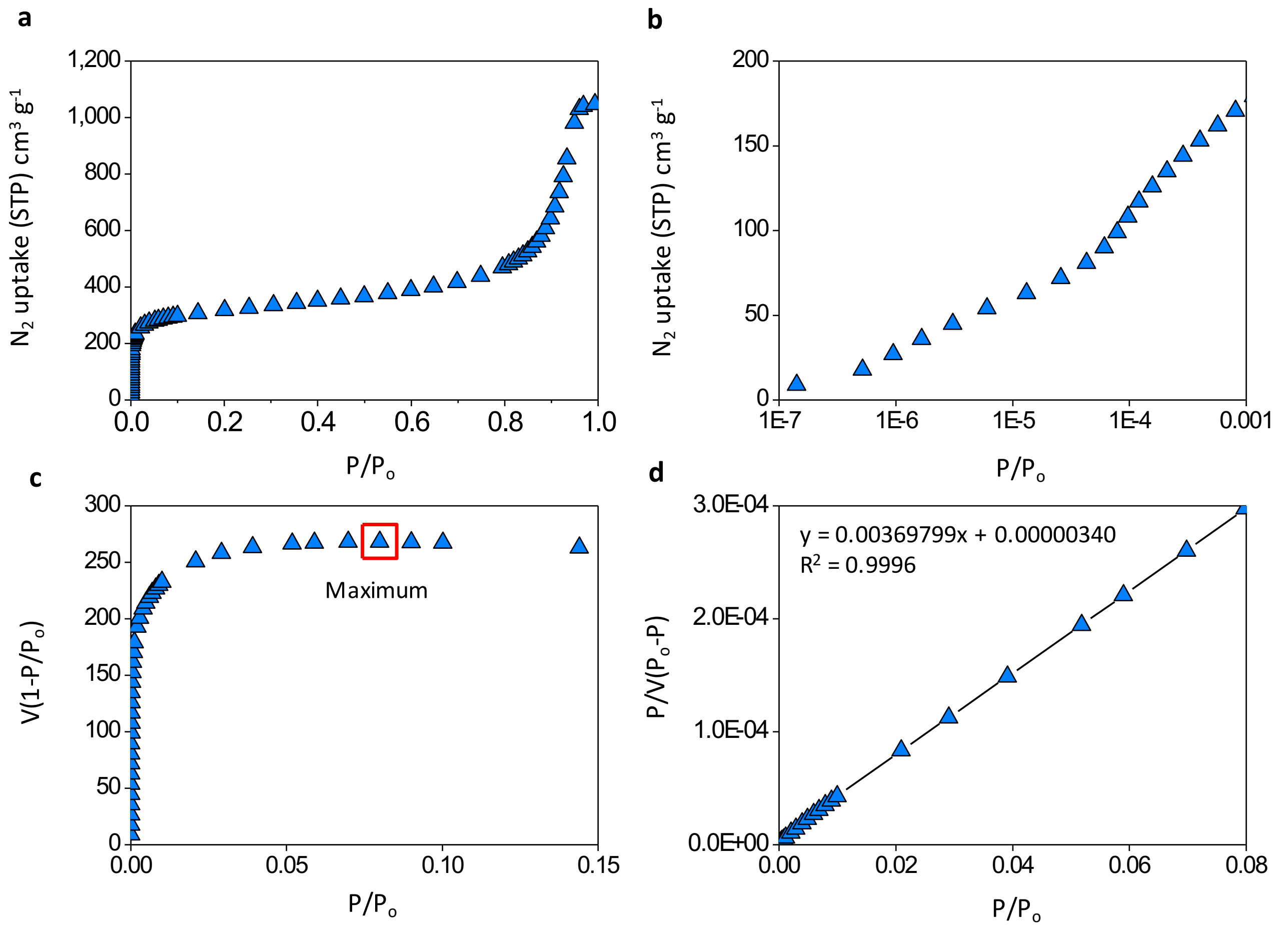

Supplementary Figure 9| N_2 adsorption isotherms and BET analysis of $_{mono}$UiO-66_A. N_2 gas adsorption isotherms were collected between 0-1 bar at 77 K. **a, b,** N_2 adsorption isotherms represented as linear and semi-log plots respectively, **c,** Determination of the maximum P/P_o (red square) using Rouquerol's consistency criteria.[10] **d,** BET representation of N_2 isotherm showing the linear range utilised in the surface area calculations for each monolith. The linear equation and R^2 value obtained from the linear fit of the data are also given.

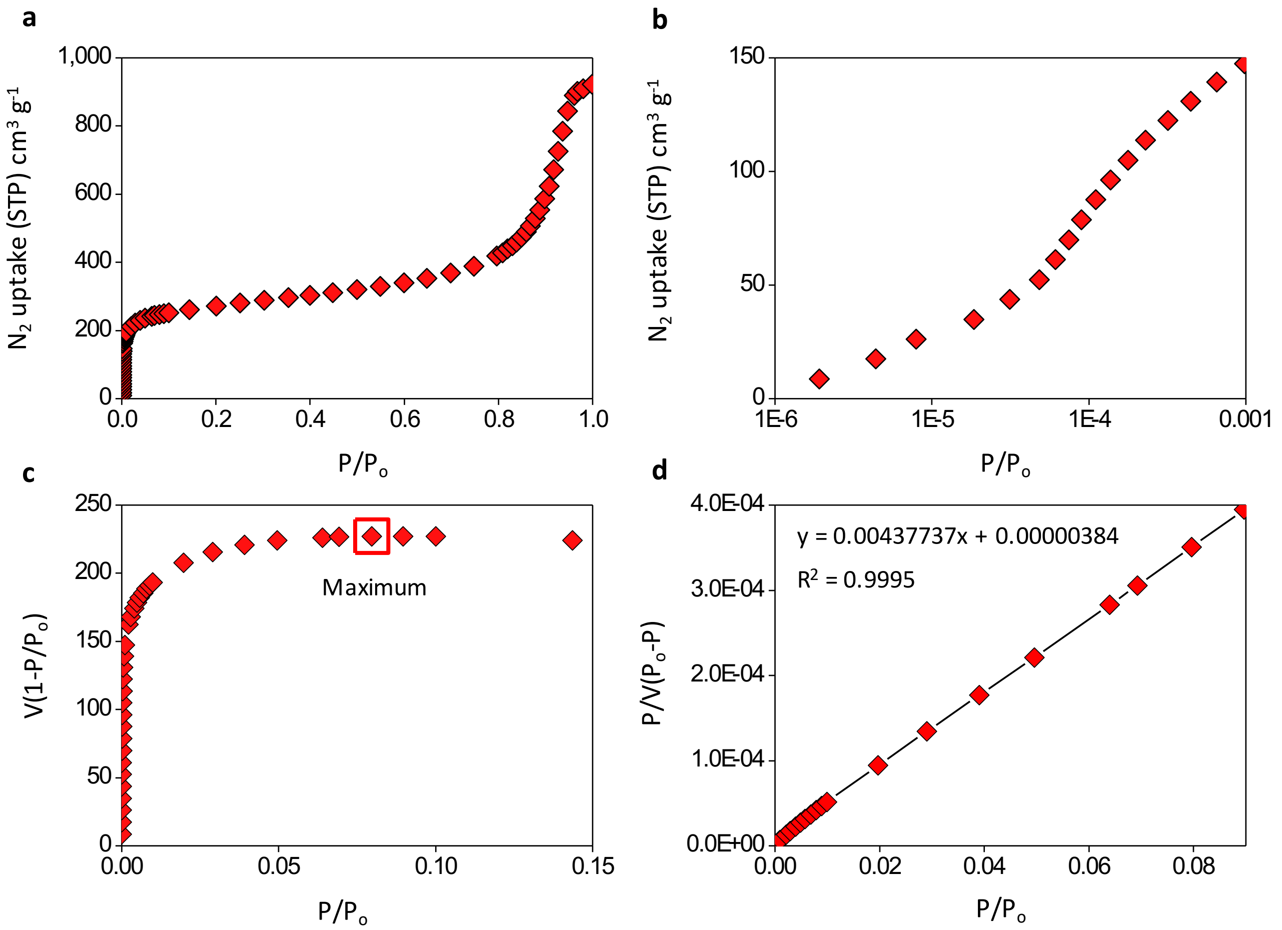

Supplementary Figure 10| N_2 adsorption isotherms and BET analysis of $_{mono}$UiO-66_B. N_2 gas adsorption isotherms were collected between 0-1 bar at 77 K. **a, b,** N_2 adsorption isotherms represented as linear and semi-log plots respectively, **c,** Determination of the maximum P/P_o (red square) using Rouquerol's consistency criteria.[10] **d,** BET representation of N_2 isotherm showing the linear range utilised in the surface area calculations for each monolith. The linear equation and R^2 value obtained from the linear fit of the data are also given.

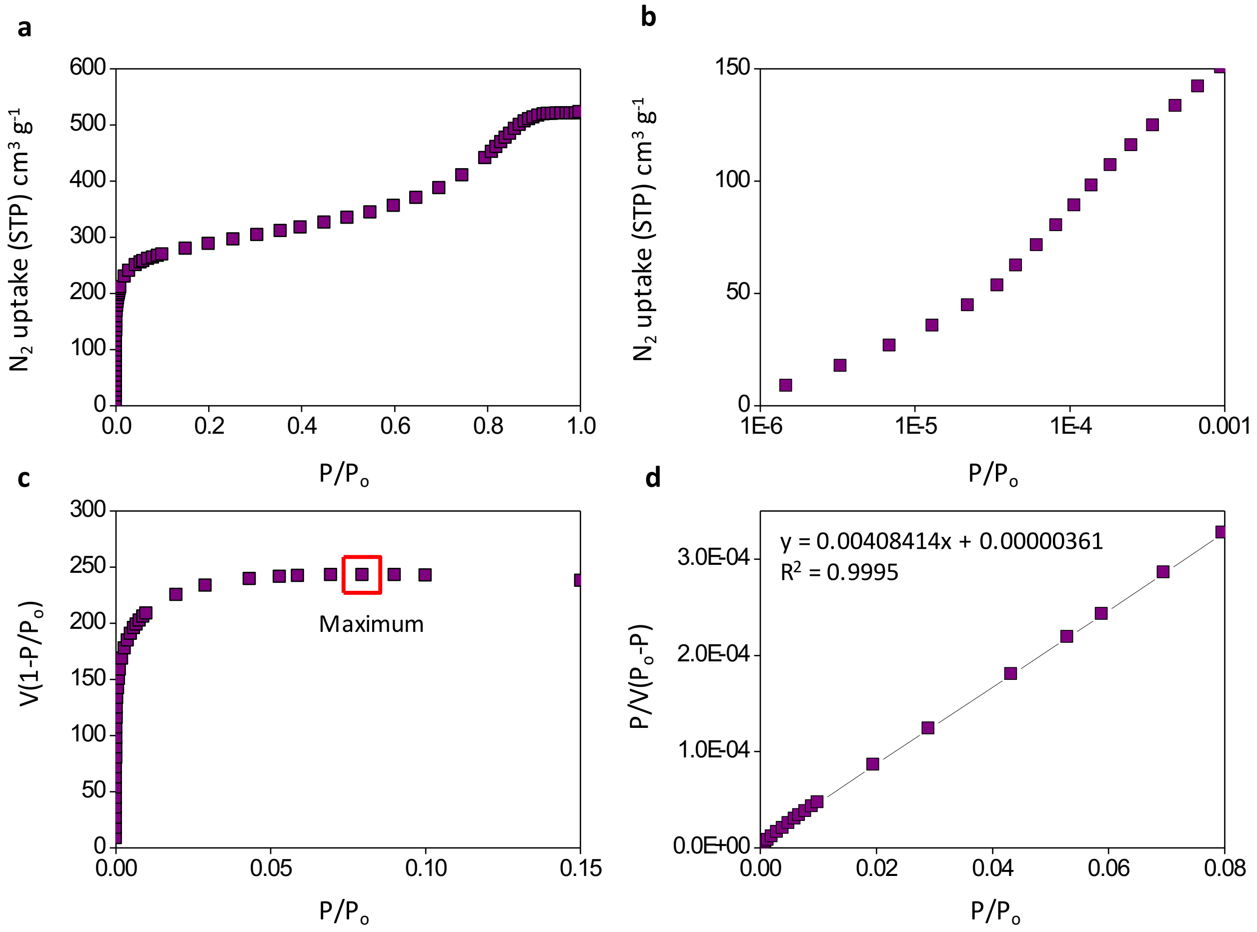

Supplementary Figure 11| N_2 adsorption isotherms and BET analysis of $_{mono}$UiO-66_C. N_2 gas adsorption isotherms were collected between 0-1 bar at 77 K. **a, b,** N_2 adsorption isotherms represented as linear and semi-log plots respectively, **c,** Determination of the maximum P/P_o (red square) using Rouquerol's consistency criteria.[10] **d,** BET representation of N_2 isotherm showing the linear range utilised in the surface area calculations for each monolith. The linear equation and R^2 value obtained from the linear fit of the data are also given.

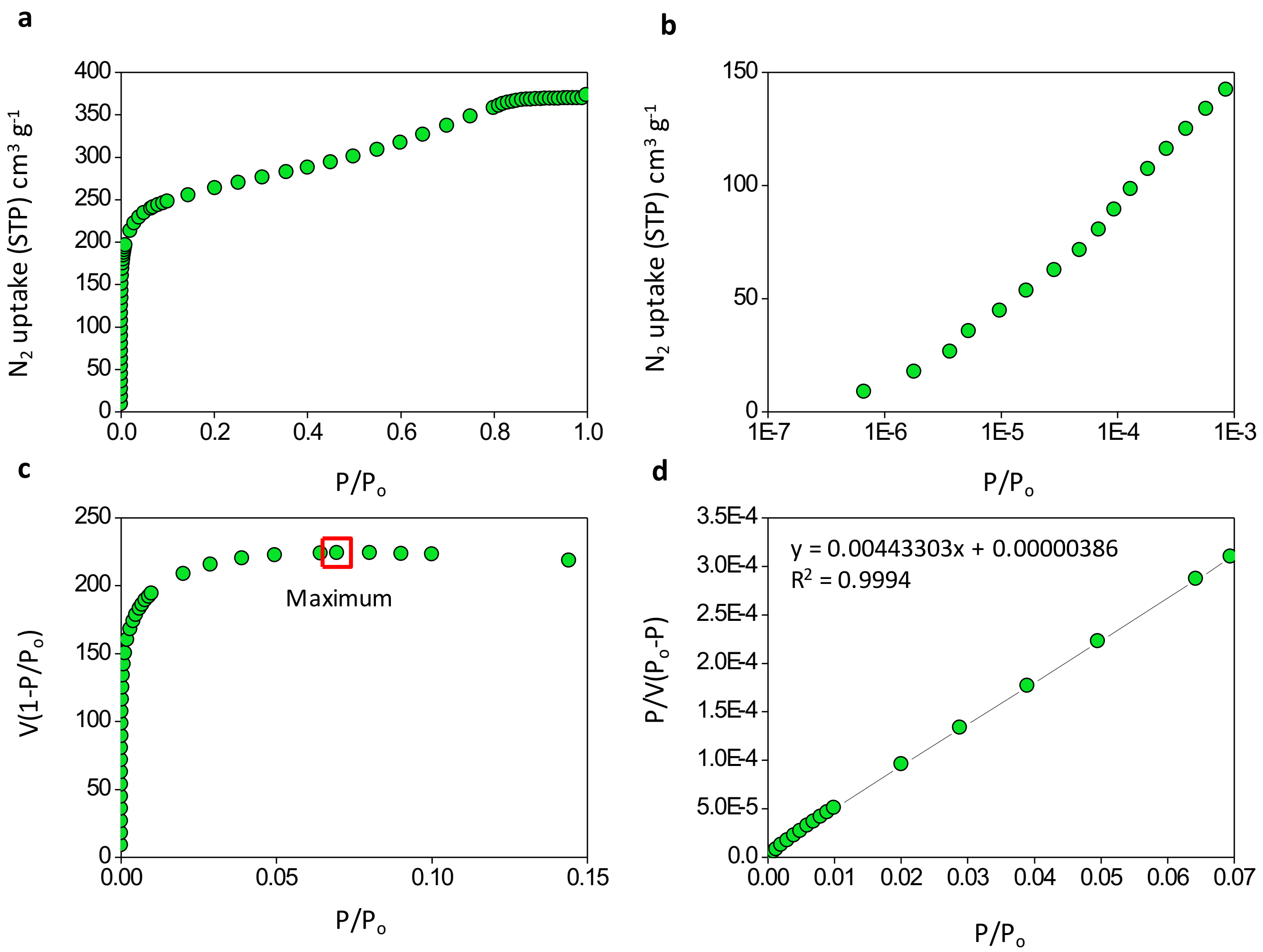

Supplementary Figure 12| N_2 adsorption isotherms and BET analysis of $_{mono}$UiO-66_D. N_2 gas adsorption isotherms were collected between 0-1 bar at 77 K. **a, b,** N_2 adsorption isotherms represented as linear and semi-log plots respectively, **c,** Determination of the maximum P/P_o (red square) using Rouquerol's consistency criteria.[10] **d,** BET representation of N_2 isotherm showing the linear range utilised in the surface area calculations for each monolith. The linear equation and R^2 value obtained from the linear fit of the data are also given.

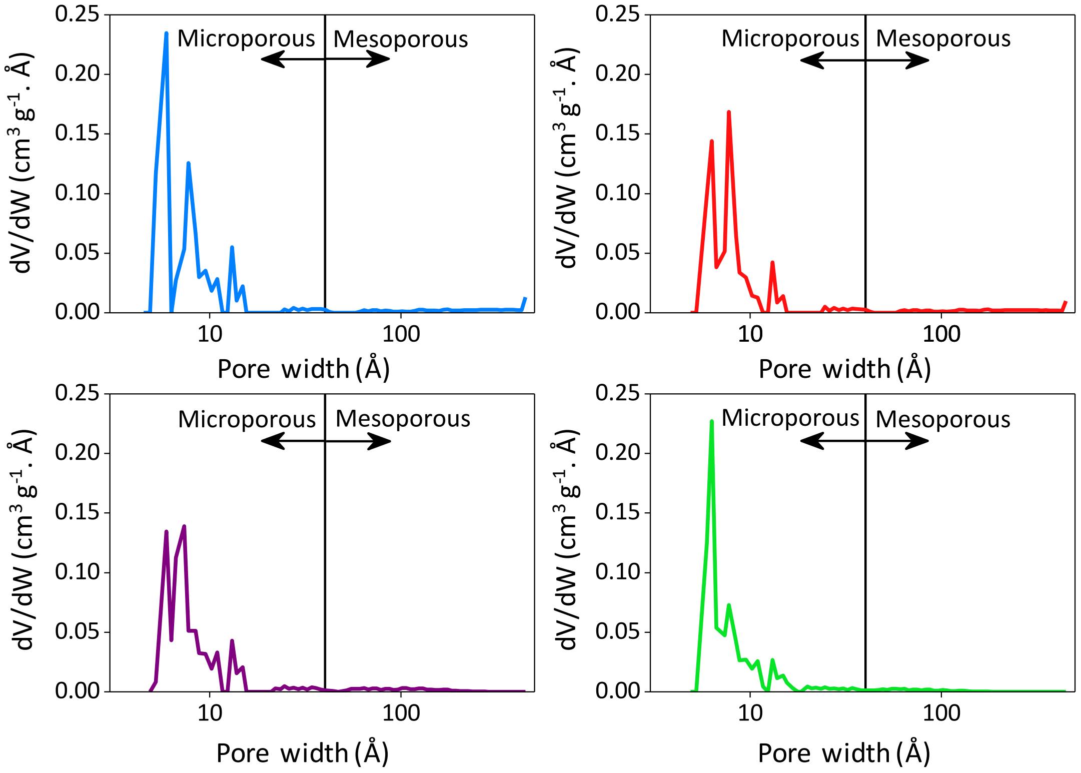

Supplementary Figure 13| NLDFT pore size distributions of $_{mono}$UiO-66. a, The distribution of micro- and mesopore width across monolith samples: UiO-66_A (blue); UiO-66_B (red); UiO-66_C (purple) and UiO-66_D (green), as obtained from Tarazona Non-Local Density Functional Theory (NLDFT) model analysis of N_2 isotherm data (Supplementary Figs. 8-12).

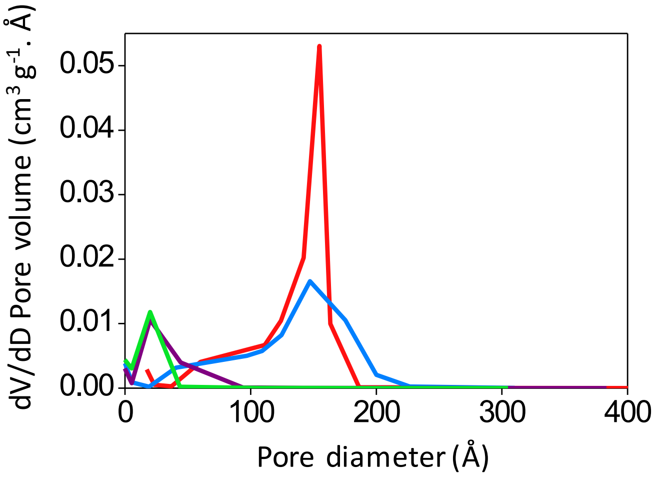

Supplementary Figure 14| BJH pore size distributions of $_{mono}$UiO-66. Distribution of mesopore diameter across monolith samples: UiO-66_A (blue); UiO-66_B (red); UiO-66_C (purple) and UiO-66_D (green), as obtained from Barrett-Joyner-Halenda (BJH) model analysis of N_2 isotherm data (Supplementary Figs. 8-12).

Supplementary Notes 2| Computational details. The simulated adsorption of N_2, CH_4, and CO_2 was investigated using grand canonical Monte Carlo (GCMC) simulations performed with the multi-purpose simulation package RASPA.[11] We used the standard Lennard-Jones (LJ) 12-6 plus Coulomb potential to model the interactions between guest-guest and guest-host atoms. The LJ parameters for the framework atoms were obtained from the Universal Force Field (UFF).[12]

The periodic long-range electrostatic interactions were calculated by the Ewald summation technique and the extended charge equilibration (EQeq) scheme[13] was used to estimate partial atomic charges in the framework.

CH_4 was modelled with a single interaction centre, while CO_2 was described with a three-interaction sites linear rigid model each site corresponding to a carbon or oxygen atom with bond length of 1.16 Å (C - O). The N_2 molecule was described with a three-interaction sites linear rigid model (two sites corresponding to the nitrogen atoms and a central one describing the centre of mass) with bond length of 0.55 Å (N_N_2 - N_com). The gas molecules Lennard-Jones parameters were modelled using the TraPPE potential (N_2,[14] CO_2[14] and CH_4[15]).

The Lorentz-Berthelot mixing rules were employed to calculate fluid/solid LJ parameters. To calculate the gas-phase fugacity, we used the Peng-Robinson equation of state.

Supplementary Table 3| Lennard-Jones parameters for UiO-66,[12] N_2,[14] CO_2[14] and CH_4.[15]

Atom	σ [Å]	ε/k [K]	q [e]
		UFF	
C_{MOF}	3.431	52.836	
O_{MOF}	3.119	30.192	
H_{MOF}	2.572	22.141	
Zr_{MOF}	2.784	34.721	
		TraPPE	
N_N_2	3.310	36	-0.482
N_com	0	0	0.964
C	2.800	27	0.700
O	3.050	79	-0.350
CH_4	3.730	148	0

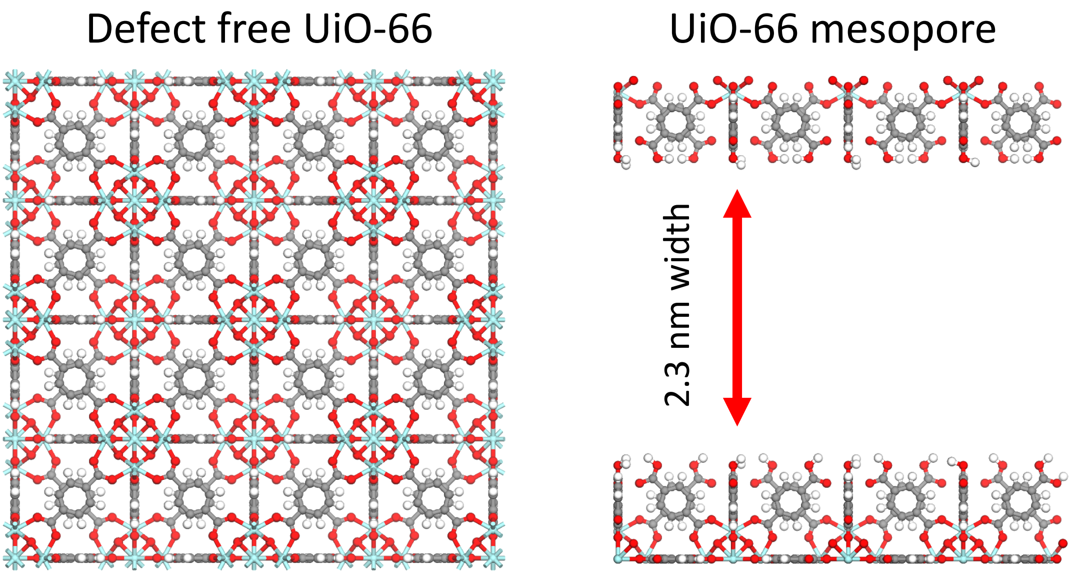

Supplementary Figure 15| Engineered mesopore defects in UiO-66. Figures showing the crystal structures of original/defect-free microporous UiO-66 (left) and engineered micro-/meso-porous UiO-66 (right) with a gap length of 2.3 nm to match the experimental N_2 isotherm of $_{mono}$UiO-66_D.

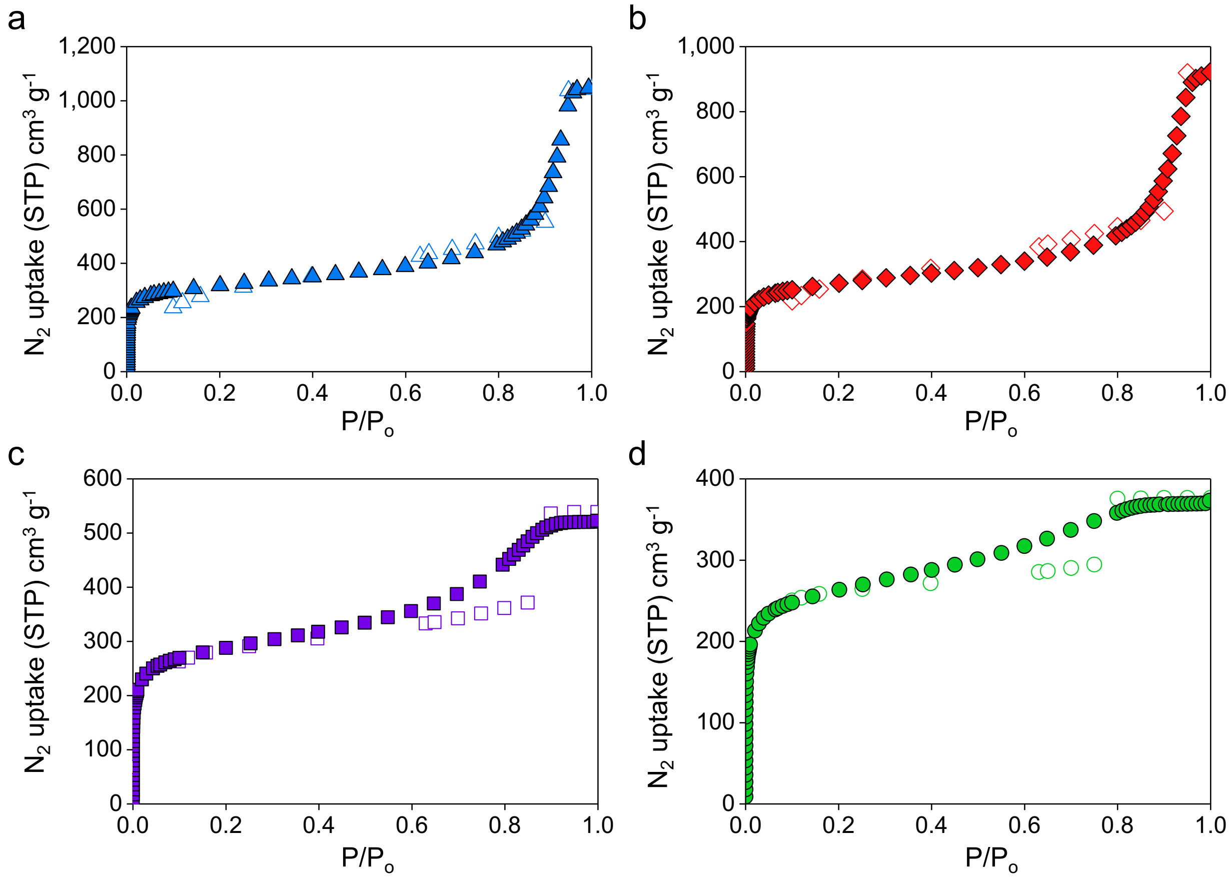

Supplementary Figure 16| Simulated N_2 isotherms for mixed micro-/mesoporous monoliths. N_2 adsorption isotherms for UiO-66 monoliths. Filled markers show the experimentally obtained isotherms while hollow markers show the computationally simulated isotherms for engineered mixed micro-/mesoporous UiO-66, **a, b,** with a gap length of 2.75 nm (to match experimental isotherms of $_{\text{mono}}$UiO-66_A and _B respectively), **c,** 2.5 nm (to match $_{\text{mono}}$UiO-66_C) and **d,** 2.3 nm (to match $_{\text{mono}}$UiO-66_D).

Supplementary Notes 3| High pressure adsorption. Adsorption and desorption isotherms for both CH_4 (0-100 bar at 298 K) and CO_2 (0-40 bar at 298 K) were experimentally obtained as excess gravimetric uptake. Gravimetric uptake (g g^{-1}) was converted to volumetric (cm^3 (STP) cm^{-3}, STP = standard temperature and pressure) uptake using the bulk density of the monolith (ρ_b, g cm^{-3}).[16]

Excess gas uptake (N_{exc}) was converted to absolute gas uptake (N_{abs}) via Supplementary equation [5] using the total pore volume of the adsorbent (V_{tot}) and the density of each gas (ρ, g cm^{-3}) at the specified pressure:[8]

$$N_{abs} = N_{exc} + \rho V_{tot} \qquad \text{[Supplementary equation 5]}$$

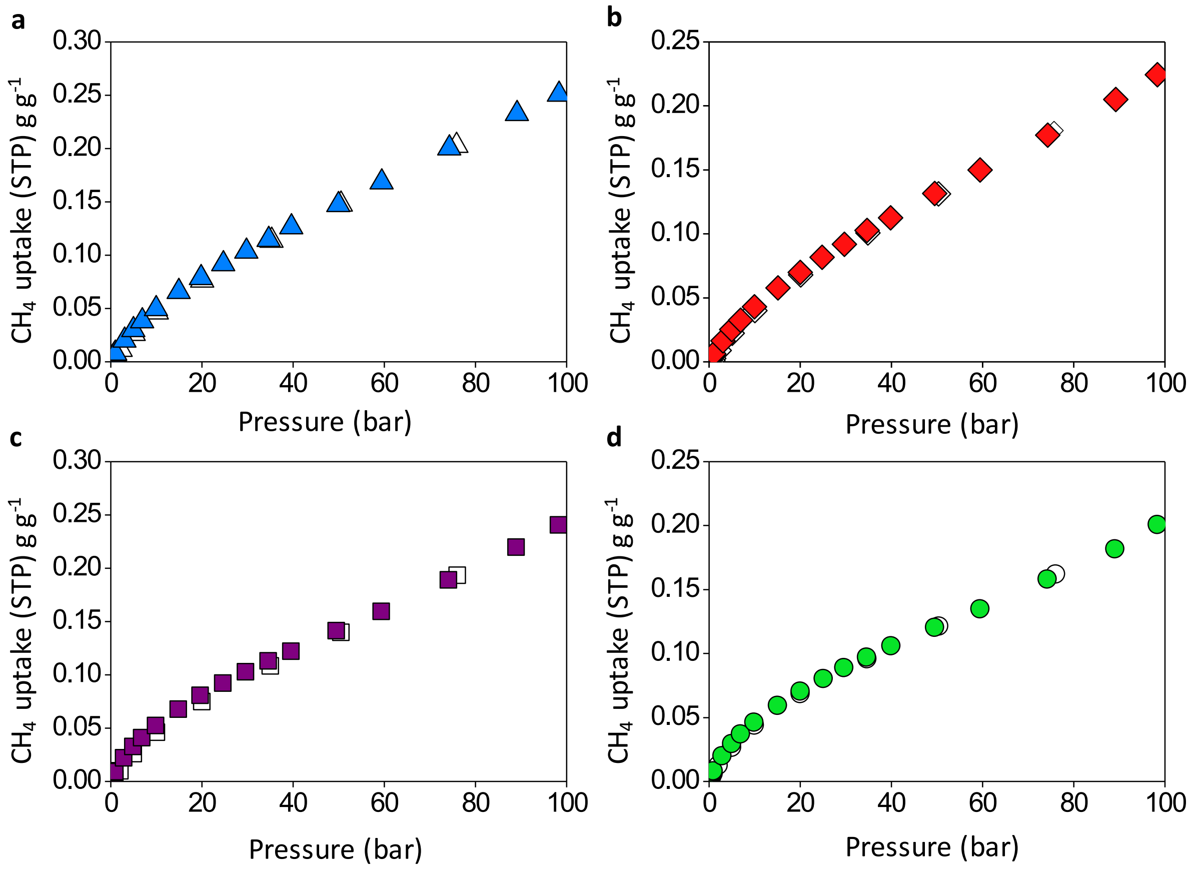

Supplementary Figure 17| CH_4 adsorption-desorption isotherms for $_{mono}$UiO-66. Gravimetric (g g^{-1}) adsorption (filled markers) and desorption (hollow markers) isotherms for **a,** UiO-66_A (blue); **b,** UiO-66_B (red); **c,** UiO-66_C (purple) and **d,** UiO-66_D (green) represented as absolute uptake between 0-100 bar methane. An absence of hysteresis was observed across all samples.

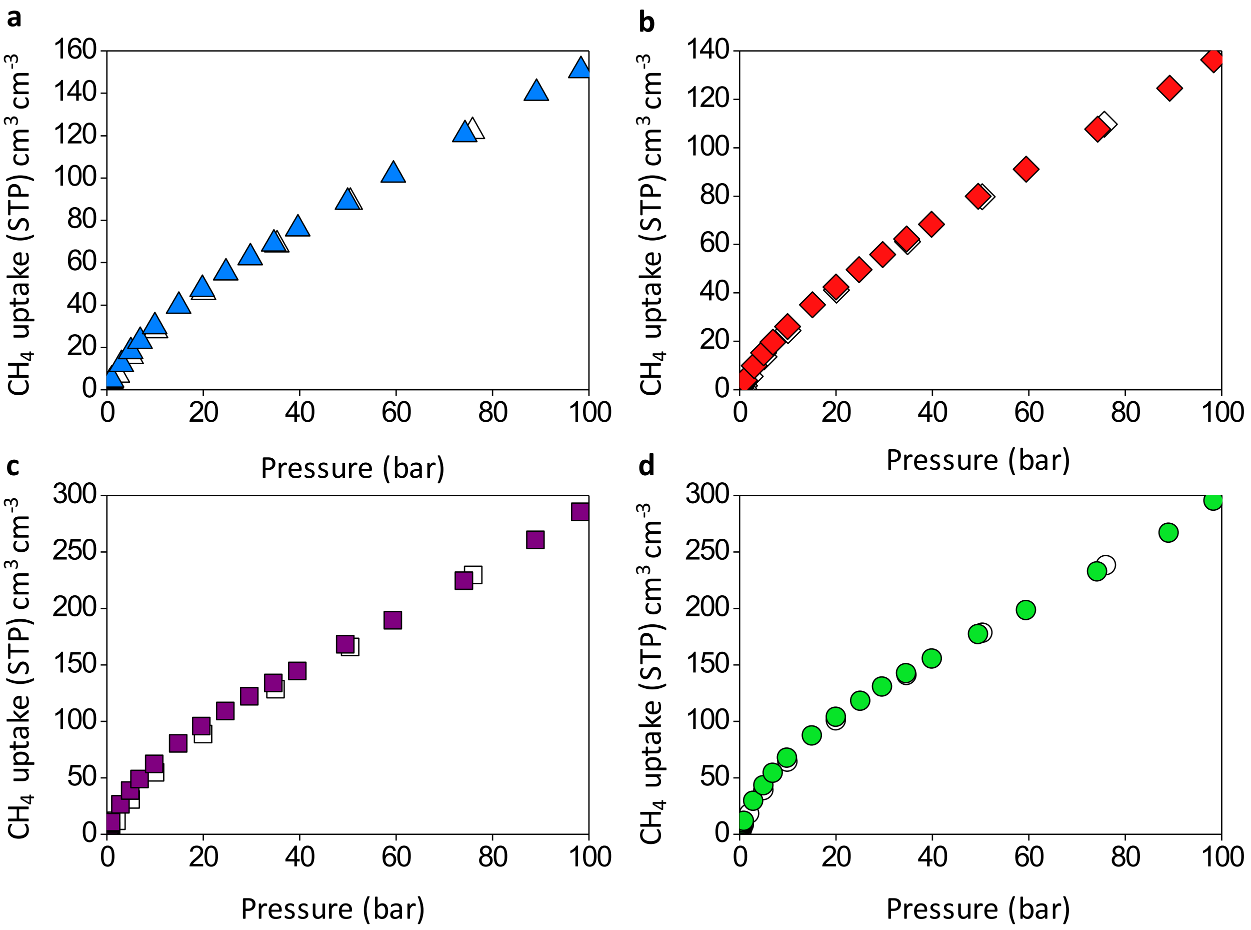

Supplementary Figure 18| CH_4 adsorption-desorption isotherms for $_{mono}$UiO-66. Volumetric (cm^3 (STP) cm^{-3}) adsorption (filled markers) and desorption (hollow markers) isotherms for **a,** UiO-66_A (blue); **b,** UiO-66_B (red); **c,** UiO-66_C (purple) and **d,** UiO-66_D (green) represented as absolute uptake between 0-100 bar methane. An absence of hysteresis was observed across all samples.

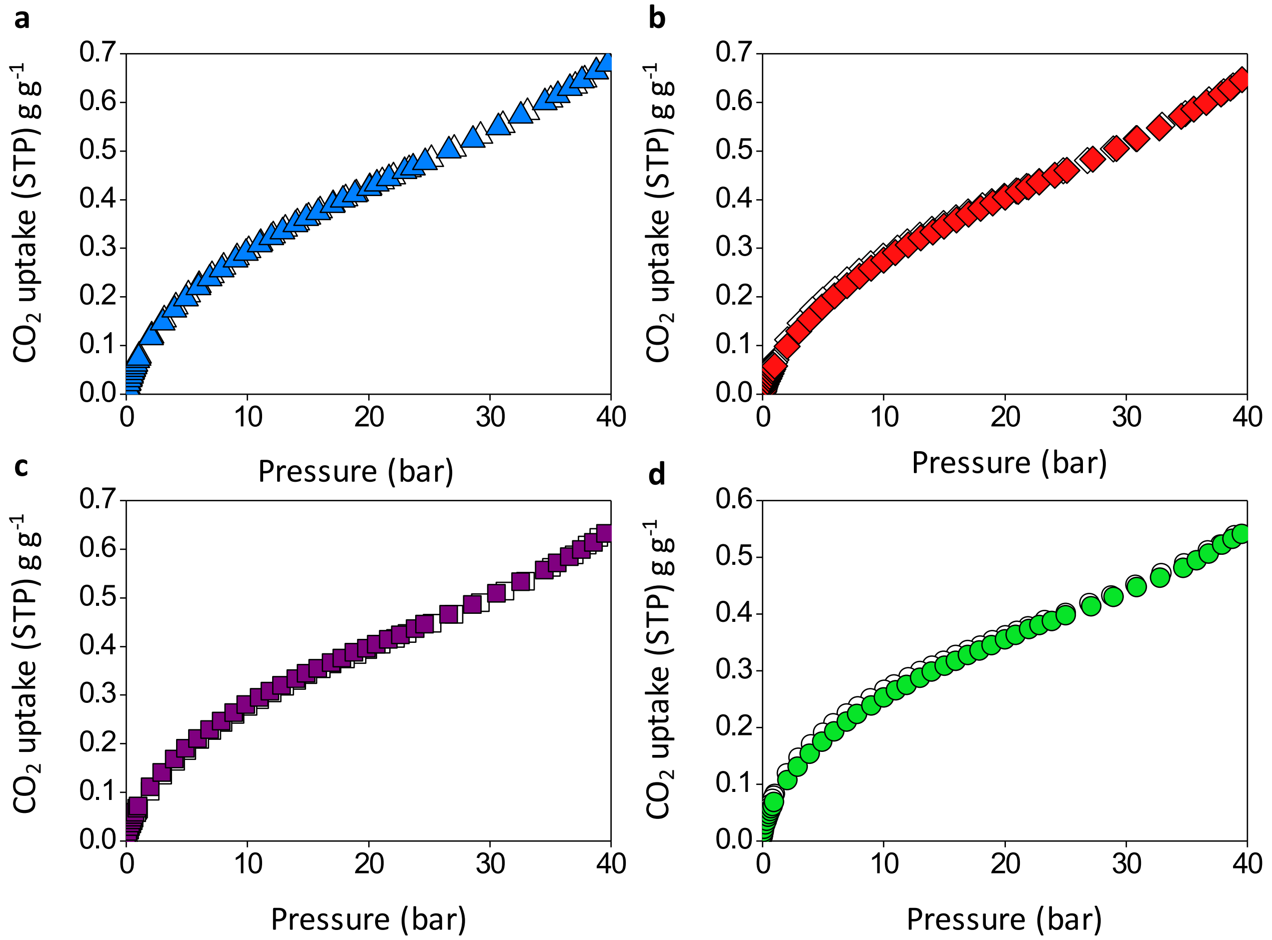

Supplementary Figure 19| CO_2 adsorption-desorption isotherms for $_{mono}$UiO-66. Gravimetric (g g^{-1}) adsorption (filled markers) and desorption (hollow markers) isotherms for **a,** UiO-66_A (blue); **b,** UiO-66_B (red); **c,** UiO-66_C (purple) and **d,** UiO-66_D (green) represented as absolute uptake between 0-40 bar carbon dioxide. An absence of hysteresis was observed across all samples.

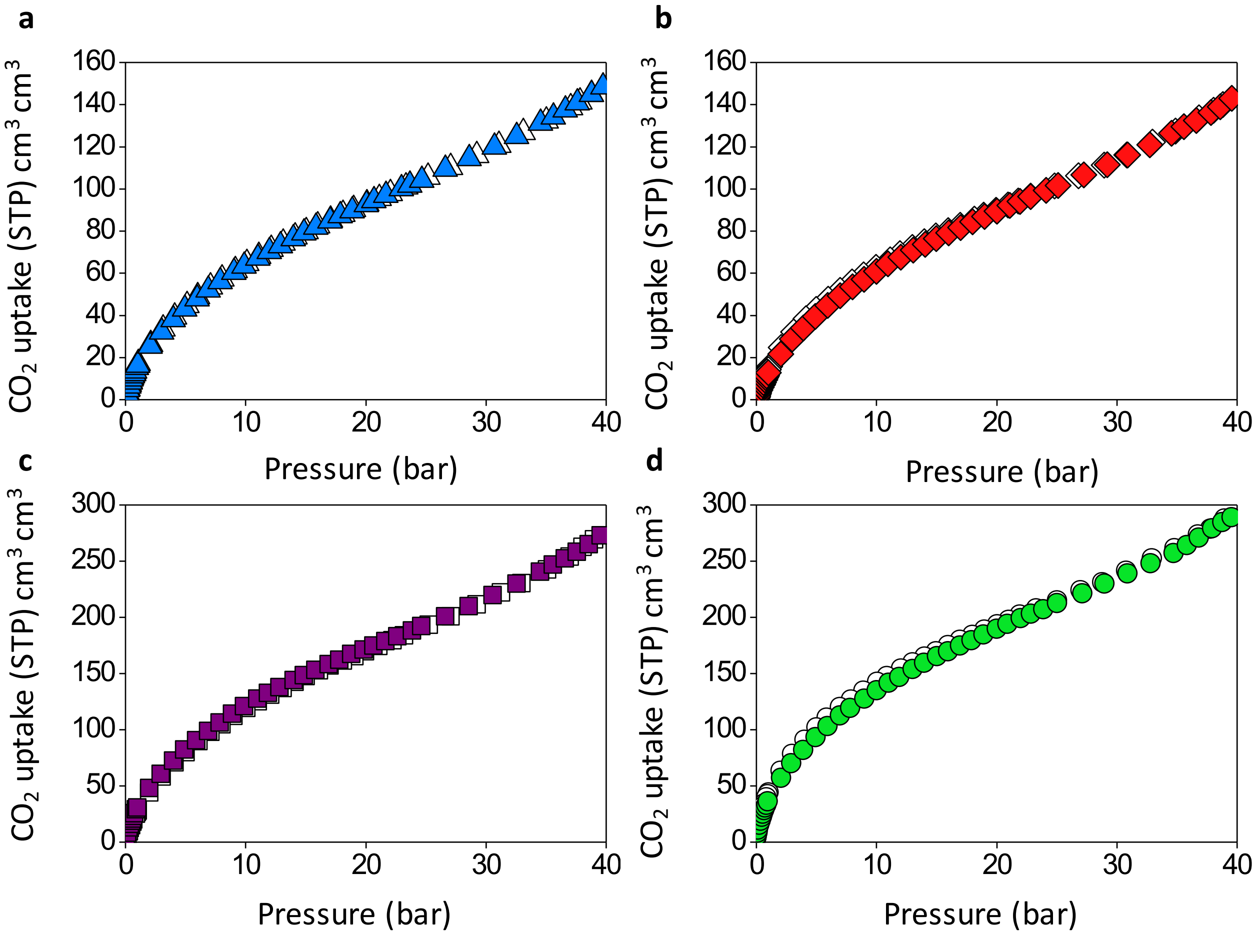

Supplementary Figure 20| CO_2 adsorption-desorption isotherms for $_{mono}$UiO-66. Volumetric (cm^3 (STP) cm^{-3}) adsorption (filled markers) and desorption (hollow markers) isotherms for **a,** UiO-66_A (blue); **b,** UiO-66_B (red); **c,** UiO-66_C (purple) and **d,** UiO-66_D (green) represented as absolute uptake between 0-40 bar carbon dioxide. An absence of hysteresis was observed across all samples.

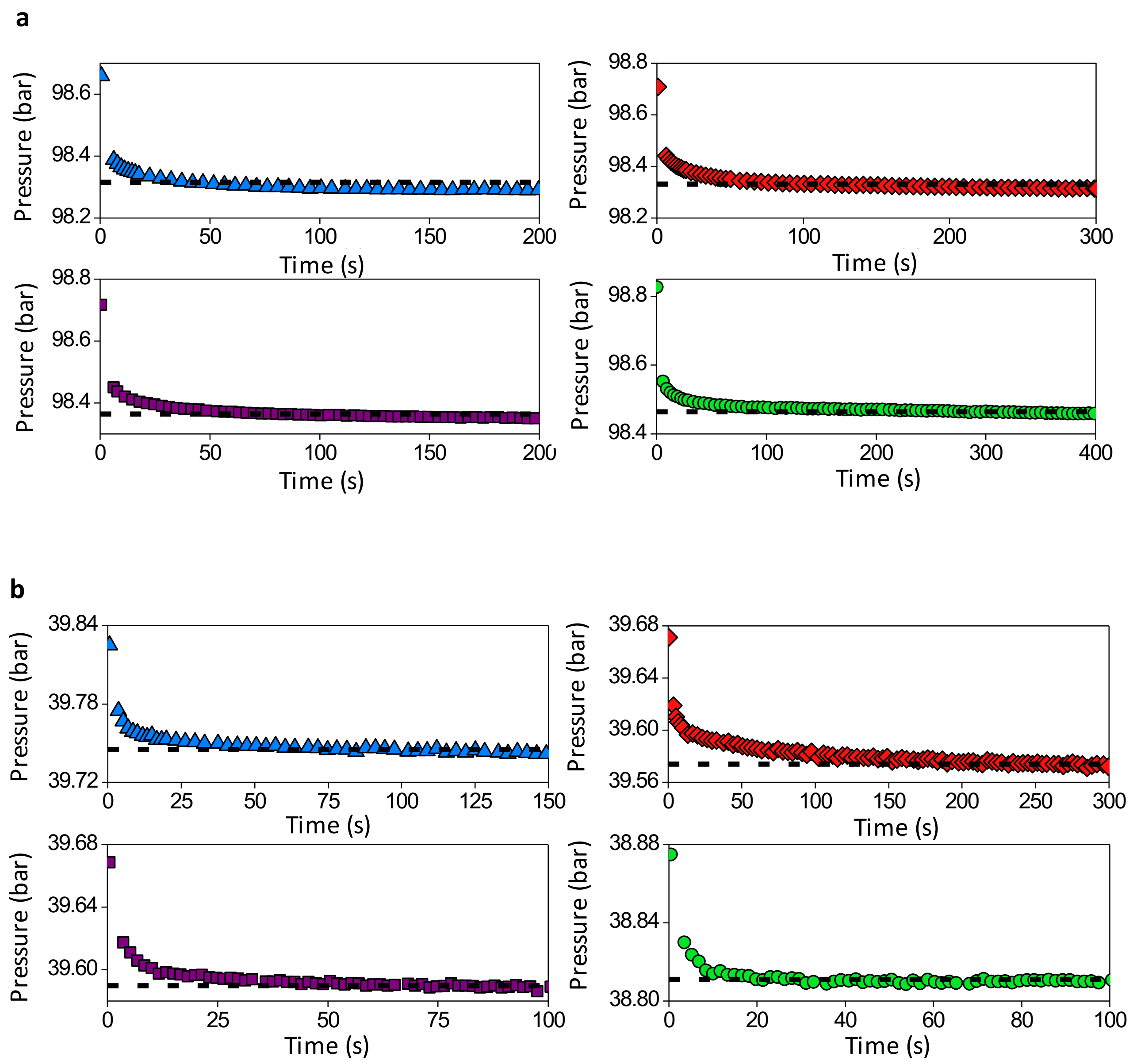

Supplementary Figure 21| Gas uptake kinetics for $_{mono}$UiO-66. Gas uptake kinetics for $_{mono}$UiO-66_A-D were recorded by measuring the time taken to reach 95 % equilibrium pressure after gas dosing. Decay of pressure in UiO-66_A (blue), UiO-66_B (red), UiO-66_C (purple) and UiO-66_D (green) for **a,** methane at ca. 100 bar and **b,** carbon dioxide at ca. 40 bar. Dashed black line indicates the pressure at which 95 % uptake equilibrium was obtained. The gas adsorption kinetics in UiO-66_A-D are comparable to the fast kinetics recently reported for $_{mono}$HKUST-1 (200 s for CH_4).[8]

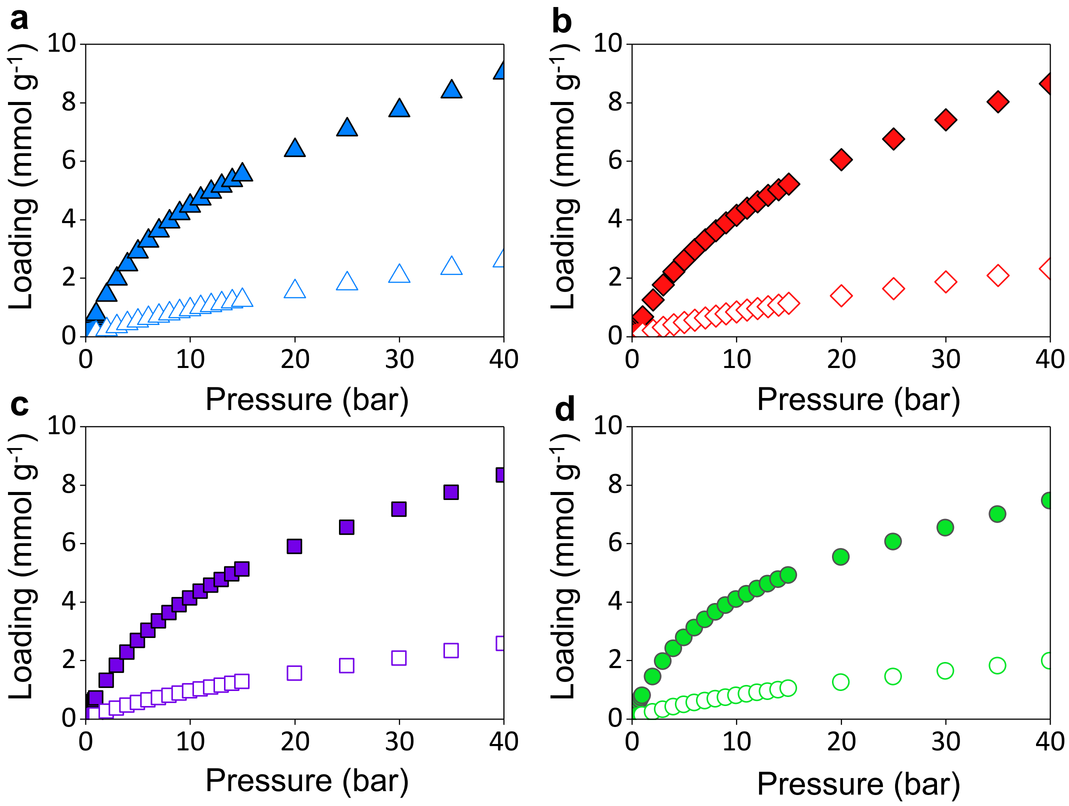

Supplementary Figure 22| Equimolar binary CO_2 and CH_4 isotherms for $_{mono}$UiO-66. Binary isotherms of CO_2 (filled marker) and CH_4 (hollow marker) uptake in monoliths **a,** UiO-66_A (blue); **b,** UiO-66_B (red); UiO-66_C (purple) and UiO-66_D (green). Adsorption data for a 50:50 gas mixture was calculated from the experimental pure-component adsorption isotherms of each pure gas at 298 K (Supplementary Figs. 17-20) using IAST.[17,18] The experimental isotherms were previously fitted to an analytical model: the BET model for CO_2 isotherms and quadratic model for CH_4 isotherms.

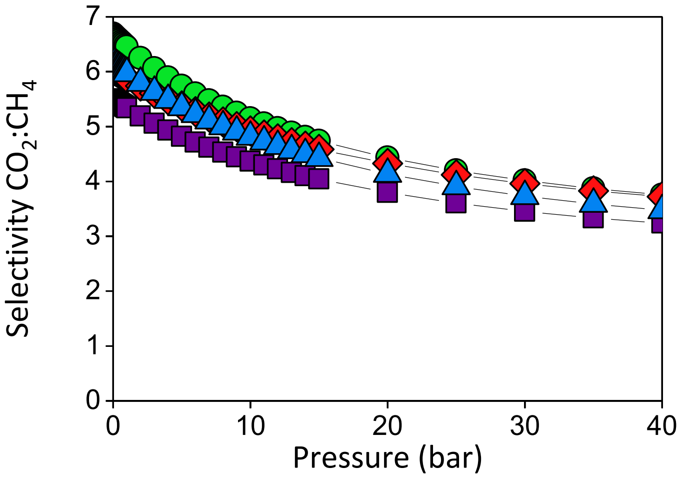

Supplementary Figure 23| Gas mixture adsorption selectivity for $_{mono}$UiO-66. CO_2:CH_4 uptake selectivity for monoliths UiO-66_A (blue), UiO-66_B (red), UiO-66_C (purple) and UiO-66_D (green). Selectivity data for a 50:50 gas mixture was calculated from the predicted equimolar binary isotherms (Supplementary Fig. 22). Results show similar selectivity in all cases (average CO_2:CH_4 selectivity amongst $_{mono}$UiO-66_A-D = 4.8 ± 0.3 at 10 bar), with each monolith exhibiting an exponential reduction in selectivity at increased pressure. This is consistent with previous observations of reduced membrane selectivity in polymeric and mixed-membrane matrixes at elevated pressures.[19]

Supplementary Notes 4| Simulated vs. experimental CH_4 uptake comparison. It is well reported that defects i.e. missing linkers, missing clusters and non-porous phases are commonly observed in experimentally obtained UiO-66.[20,21] The gas-adsorption isotherms simulated using the defect-free crystal structure (black square) were compared to experimentally obtained isotherms from microporous UiO-66 powder (white circle) to confirm the relevance/similarity of the simulations to experimental/non-perfect UiO-66.

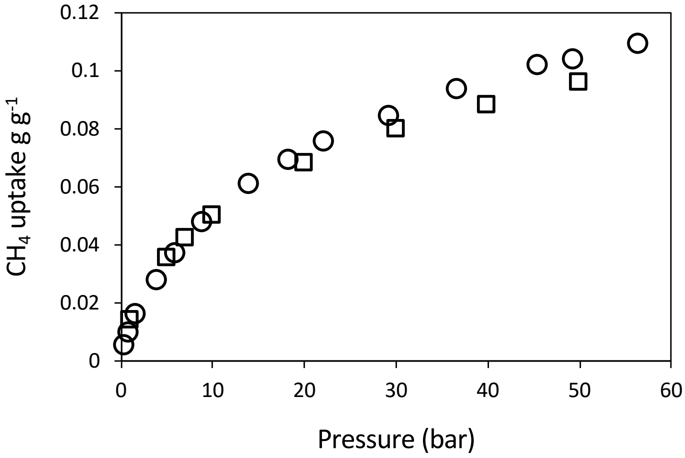

Supplementary Figure 24| CH_4 adsorption isotherms. Comparison of CH_4 isotherms in powder UiO-66 (data digitised from literature report by Wu and co-workers)[20] to isotherm simulated from the defect-free crystal structure.

Supplementary Notes 5| Snapshots and density distributions for N_2, CH_4 and CO_2 adsorption. A 2.3 nm mesopore was added into the previously defect-free crystal structure of UiO-66 to make the structure comparable to that of $_{mono}$UiO-66_D, and gas uptake was studied over a range of pressures. In each case, the exclusive adsorption of each gas into the micropores and on the mesopore walls is demonstrated at low pressure. At higher pressure, condensation in the mesopore takes place, correlating with the experimental isotherms recorded for each gas (Supplementary Figs. 8 and 17-20).

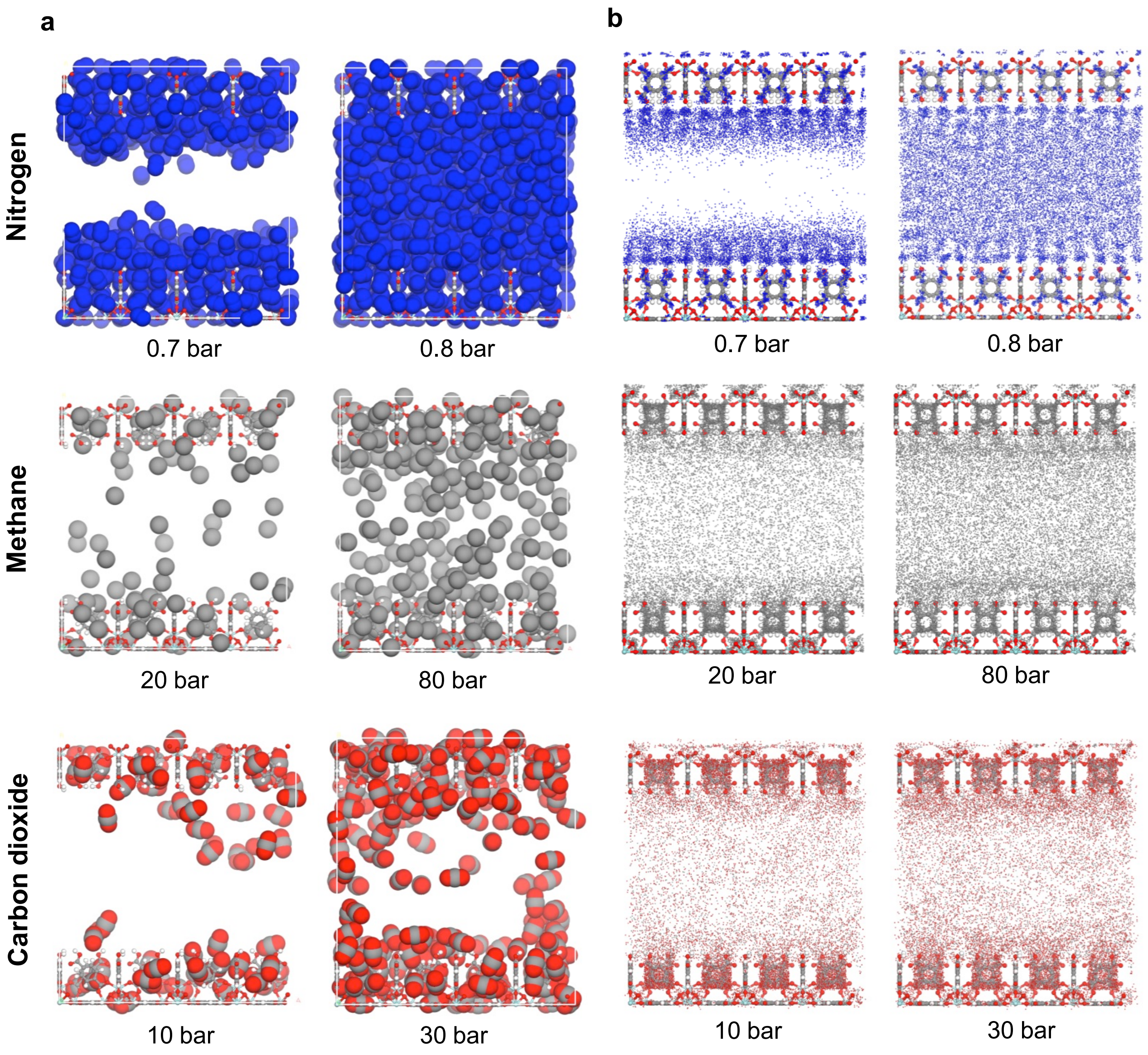

Supplementary Figure 25| Snapshots and density distributions. a, Snapshots and **b,** density distributions of nitrogen (blue), methane (grey) and carbon dioxide (grey and red) adsorption in mixed micro-/mesoporous UiO-66.

Supplementary Methods 1| Synthesis of UiO-66-NH_2 gel. 2-aminobenzene-1,4-dicarboxylic acid (1.31 g, 7.23 mmol) and zirconium(IV) oxychloride octahydrate (1.61 g, 5.0 mmol) were dissolved in N,N-dimethylformamide (30 ml, 99 %). Concentrated hydrochloric acid (1.5 ml, 37 %) and glacial acetic acid (2.0 ml) were added under vigorous stirring. The resulting solution was sealed in a 100 ml Pyrex Schott bottle and heated to 100 °C for 2 hours. This yielded UiO-66-NH_2 as a viscous yellow gel. **Synthesis of $_{mono}$UiO-66-NH_2_A-C.** N,N-dimethylformamide (50 ml, 99 %) was added to the UiO-66-NH_2 gel, as synthesised above, and vigorously mixed. The diluted UiO-66-NH_2 suspension (7.5 ml per tube) was centrifuged (3 min, 5500 rpm) and the supernatant decanted. The gel was washed, centrifuged (5500 rpm) and dried to produce a range of monoliths (Supplementary Table 4, below). The obtained monoliths were soaked in acetone (3 × 5 ml, 24 hours) and methanol (3 × 5 ml, 24 hours) and then dried at room temperature overnight. Monolith activation was by heating to 110 °C under vacuum for 8 hours.

Supplementary Table 4| Experimental conditions for $_{mono}$UiO-66-NH_2 synthesis

	Washing procedure	Centrifugation	Drying temperature (°C)
UiO-66-NH_2_A	Ethanol (3 × 30 ml)	3 × 10 min*	30
UiO-66-NH_2_B	DMF (1 × 30 ml)	1 × 10 min*	30
UiO-66-NH_2_C	DMF (1 × 30 ml)	1 × 10 min* + 1 × 180 min**	30

*Centrifugation (5500 rpm) performed after each wash to re-obtain MOF gel as sediment.

**Additional 180 min (5500 rpm) centrifugation performed on densified MOF gel after washing in DMF.

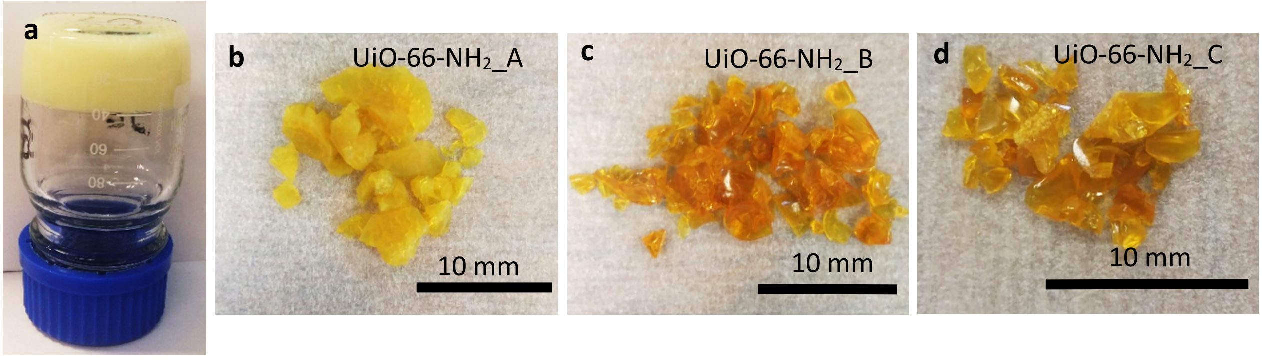

Supplementary Figure 26| Optical images of $_{mono}$UiO-66-NH$_2$. Optical images of **a**, viscous UiO-66-NH$_2$ gel, and monoliths **b**, UiO-66-NH$_2$_A, **c**, UiO-66-NH$_2$_B and **d,** UiO-66-NH$_2$_C. The optical transparency of each monolith is comparable to monoliths of UiO-66 obtained under similar experimental conditions i.e. monoliths washed in ethanol show lower optical transparency than monoliths obtained by washing in DMF.

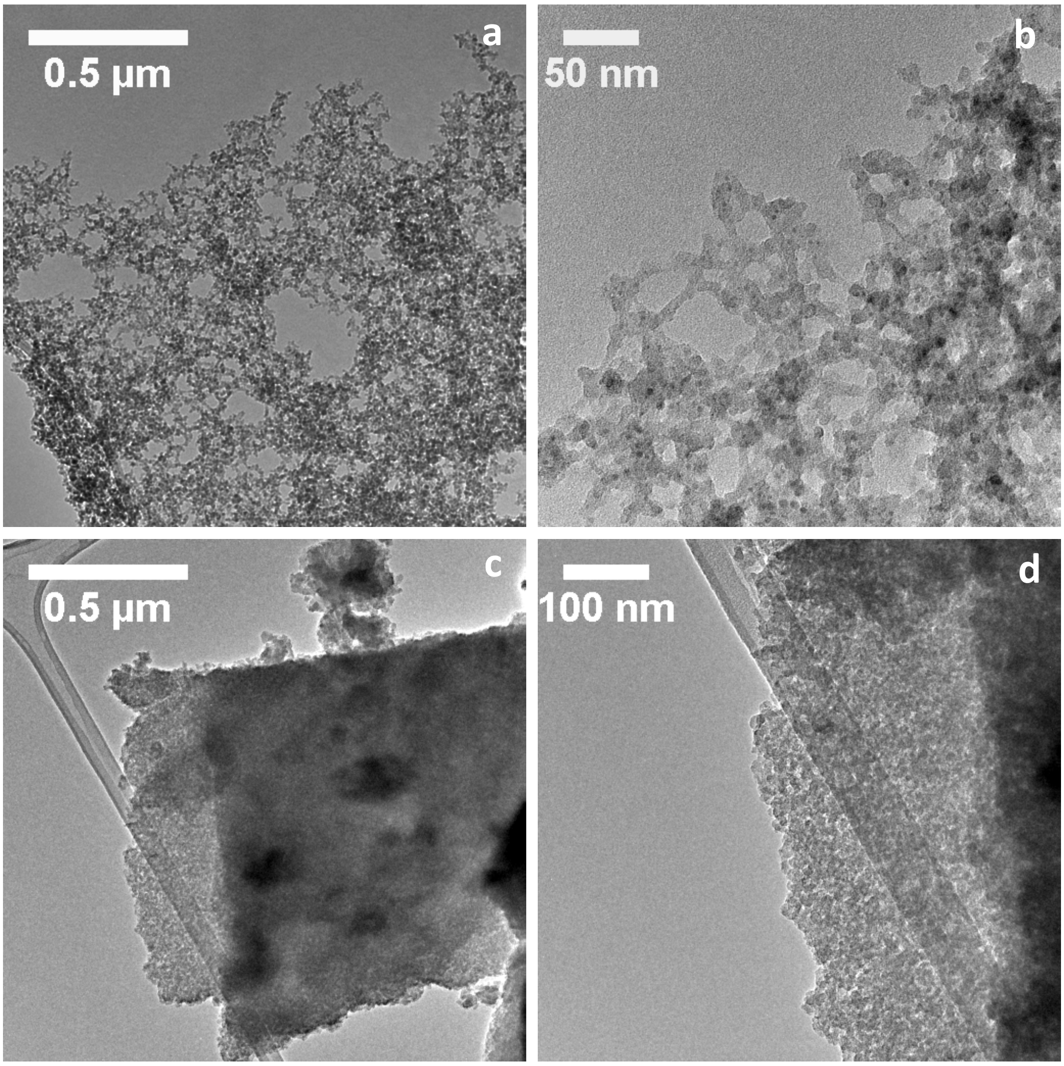

Supplementary Figure 27| $_{mono}$UiO-66-NH$_2$ electron microscopy. TEM images of **a, b,** UiO-66-NH$_2$ gel and **c, d,** $_{mono}$UiO-66-NH$_2$_C. These figures show that primary particles of ca. 10 nm diameter form a gelatinous network structure which is subsequently densified via the drying process. These observations (particle size, gelatinous macrostructure and densification upon drying) are consistent with comparable TEM observations in UiO-66 gel and $_{mono}$UiO-66 (Supplementary Figure 1). However, a secondary phase is visible as dark spots in the primary particles (Supplementary Figure 27b). We have previously reported similar defects in the MOF primary particles used to synthesise $_{mono}$HKUST-1. This was attributed to the presence of dense, non-crystalline defects within the primary particles, originating from the high speed synthesis used to obtain the small particles needed for monolith formation.[8] The presence of the amine group in 2-aminobenzene-1,4-dicarboxylic acid (UiO-66-NH$_2$ linker) increases this reagent's solubility in DMF relative to that of benzene-1,4-dicarboxylic acid (UiO-66 linker). This may facilitate UiO-66-NH$_2$ primary particle nucleation that is more rapid than that of UiO-66, the result of which is an increase in defect prevalence.

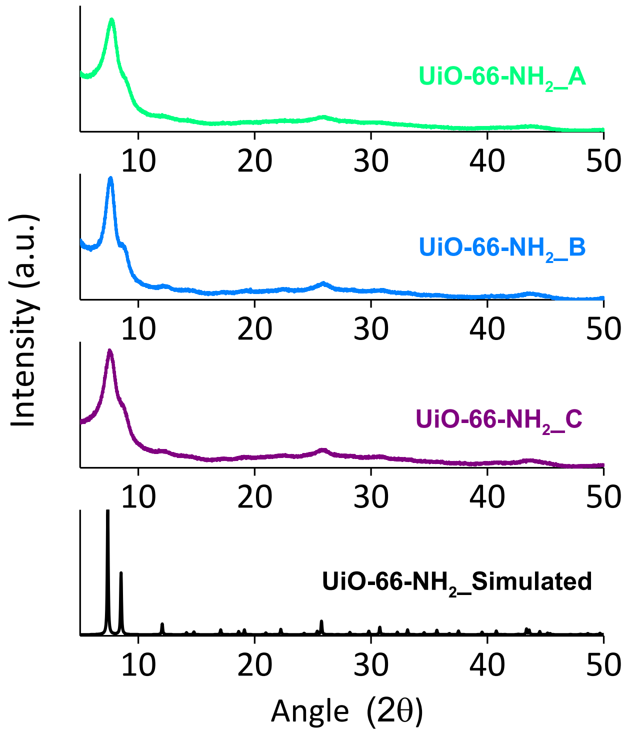

Supplementary Figure 28| X-ray diffraction $_{mono}$UiO-66-NH$_2$. Comparison of simulated XRD pattern of UiO-66-NH$_2$ generated from its crystal structure; black,[22] to XRD patterns of UiO-66-NH$_2$ monoliths: UiO-66_A (green), UiO-66_B (blue) and UiO-66_C (purple), confirming successful synthesis of the MOF in each case. For powder XRD analysis, monoliths were gently crushed using a mortar and pestle. The observation of wide peaks in the obtained XRD patterns for all experimental samples is due to line broadening characteristic of the nano size of crystalline MOF primary particles (Supplementary Fig. 27).

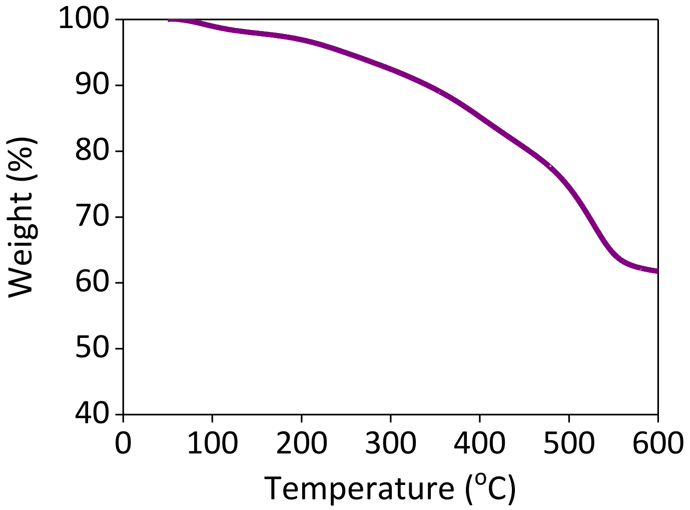

Supplementary Figure 29| Thermogravimetric analysis of $_{mono}$UiO-66-NH_2. TGA analysis of $_{mono}$UiO-66-NH_2_C showing thermal decomposition over the temperature range 50-600 °C. The recorded graph shows a gradual decomposition over the wide temperature range before ca. 550 °C. This is consistent with literature reports of UiO-66-NH_2.[23]

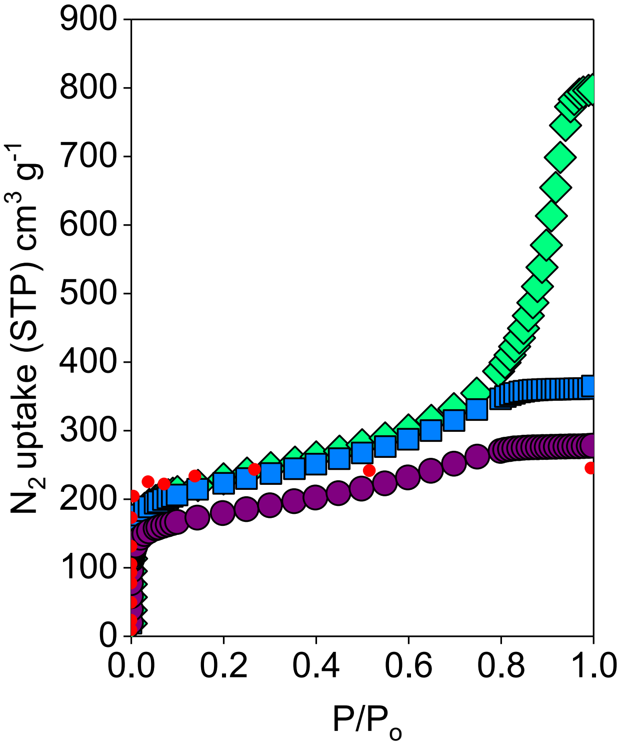

Supplementary Figure 30| N_2 adsorption isotherms for $_{mono}$UiO-66-NH_2. Linear N_2 adsorption isotherms (0-1 bar, at 77 K) for monoliths UiO-66-NH_2_A (green), UiO-66-NH_2_B (blue) and UiO-66-NH_2_C (purple). A GCMC adsorption isotherm was simulated from the crystal structure of UiO-66-NH_2 (red dots).[22]

Supplementary Table 5| BET area and pore volumes of $_{mono}$UiO-66-NH_2. BET area (S_{BET}), micropore volume (W_o) and total pore volume (V_{Tot}) of monoliths UiO-66-NH_2_A-C. Surface area was calculated by applying Rouquerol's consistency criteria to the experimentally obtained N_2 adsorption isotherms (Supplementary Figs. 31 - 33).[10]

	S_{BET} ($m^2\ g^{-1}$)	W_o* ($cm^3\ g^{-1}$)	V_{tot}† ($cm^3\ g^{-1}$)
UiO-66-NH_2_A	841	0.33	1.23
UiO-66-NH_2_B	822	0.32	0.56
UiO-66-NH_2_C	665	0.26	0.43

*Obtained at $P/P_o = 0.1$ †Obtained at $P/P_o = 0.99$

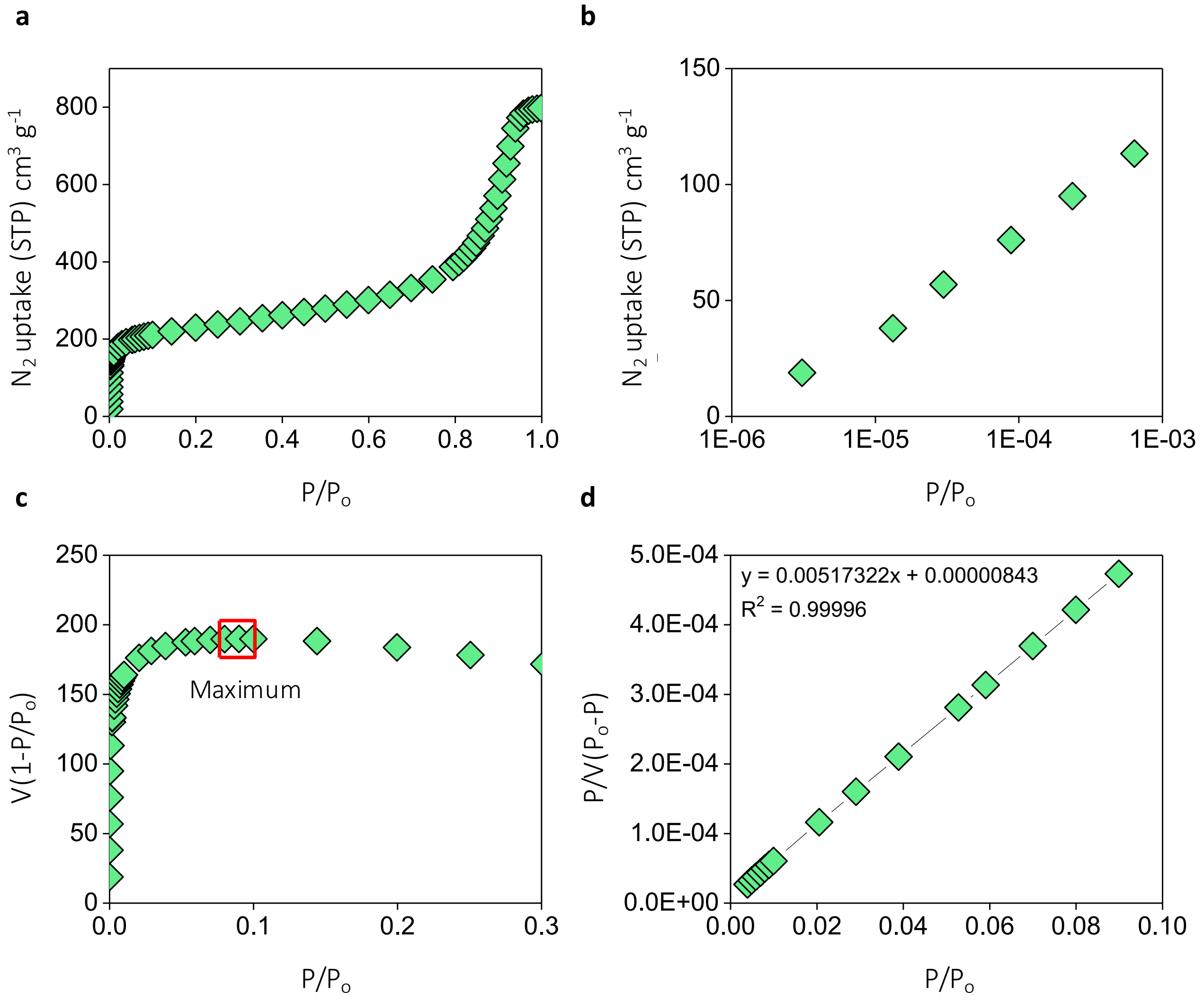

Supplementary Figure 31| N_2 adsorption isotherms and BET analysis of $_{mono}$UiO-66-NH_2_A. a, b, Linear and semi-log plot respectively of N_2 adsorption isotherms at 77 K; **c,** determination of the maximum P/P_o using Rouquerol's consistency criteria;[10] and **d,** BET representation of N_2 isotherms showing the linear range utilised in surface area calculations for each monolith. The linear equation and R^2 value obtained from the linear fit to the data are given.

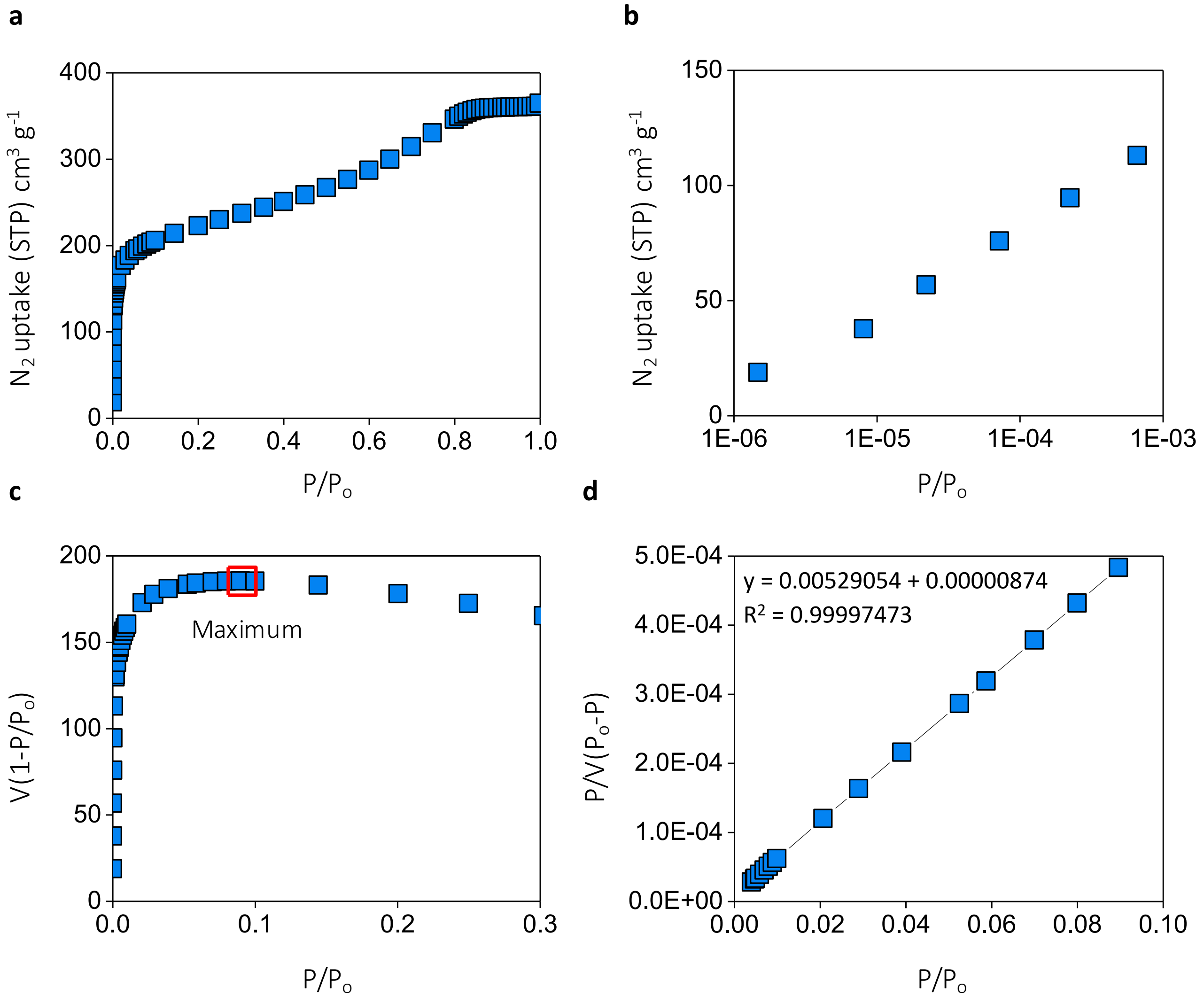

Supplementary Figure 32| N_2 adsorption isotherms and BET analysis of $_{mono}$UiO-66-NH_2_B. a a, b, Linear and semi-log plot respectively of N_2 adsorption isotherms at 77 K; **c,** determination of the maximum P/P_o using Rouquerol's consistency criteria;[10] and **d,** BET representation of N_2 isotherms showing the linear range utilised in surface area calculations for each monolith. The linear equation and R^2 value obtained from the linear fit to the data are given.

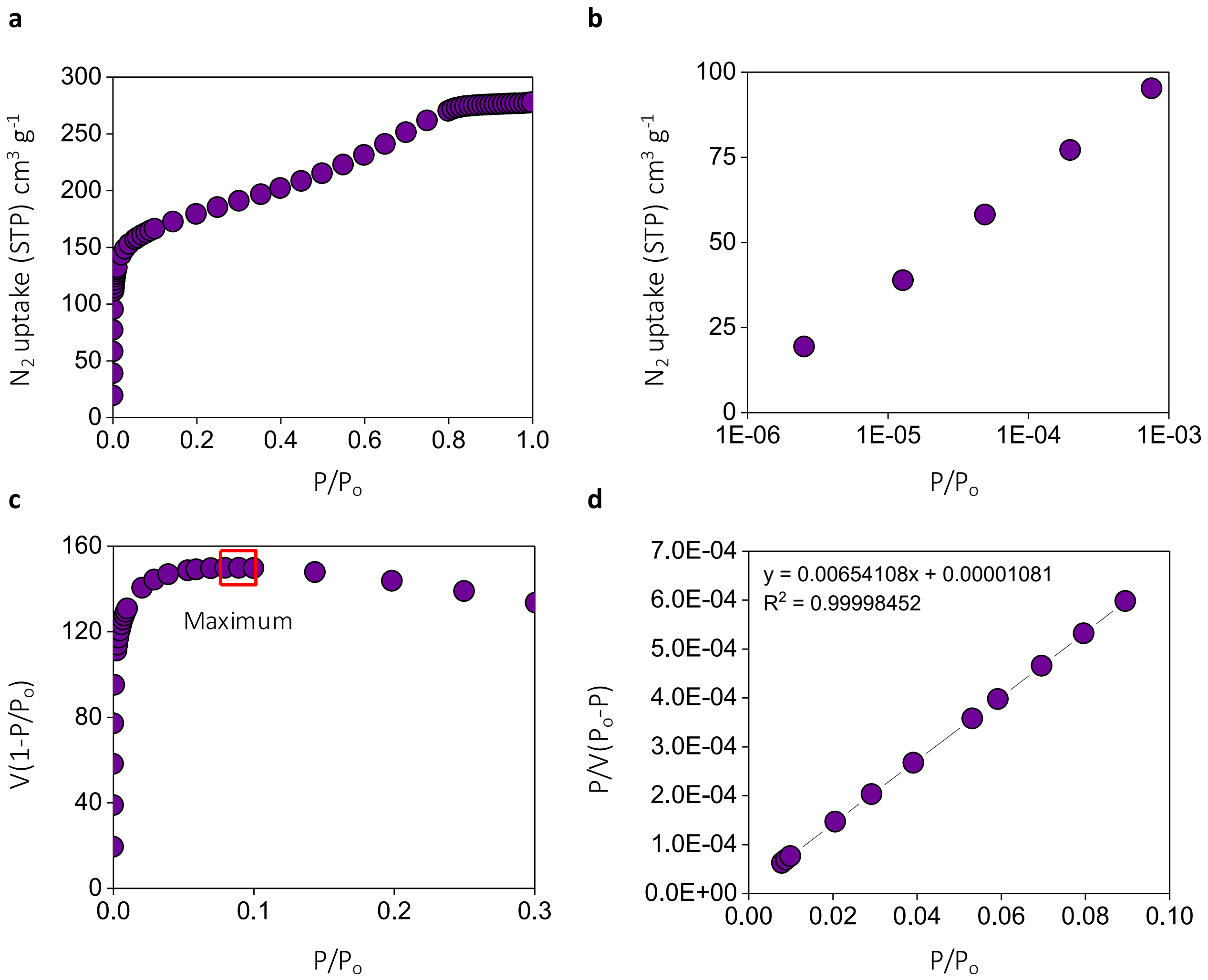

Supplementary Figure 33| N_2 adsorption isotherms and BET analysis of $_{mono}$UiO-66-NH_2_C. a, b, Linear and semi-log plot respectively of N_2 adsorption isotherms at 77 K; **c,** determination of the maximum P/P_o using Rouquerol's consistency criteria;[10] and **d,** BET representation of N_2 isotherms showing the linear range utilised in surface area calculations for each monolith. The linear equation and R^2 value obtained from the linear fit to the data are given.

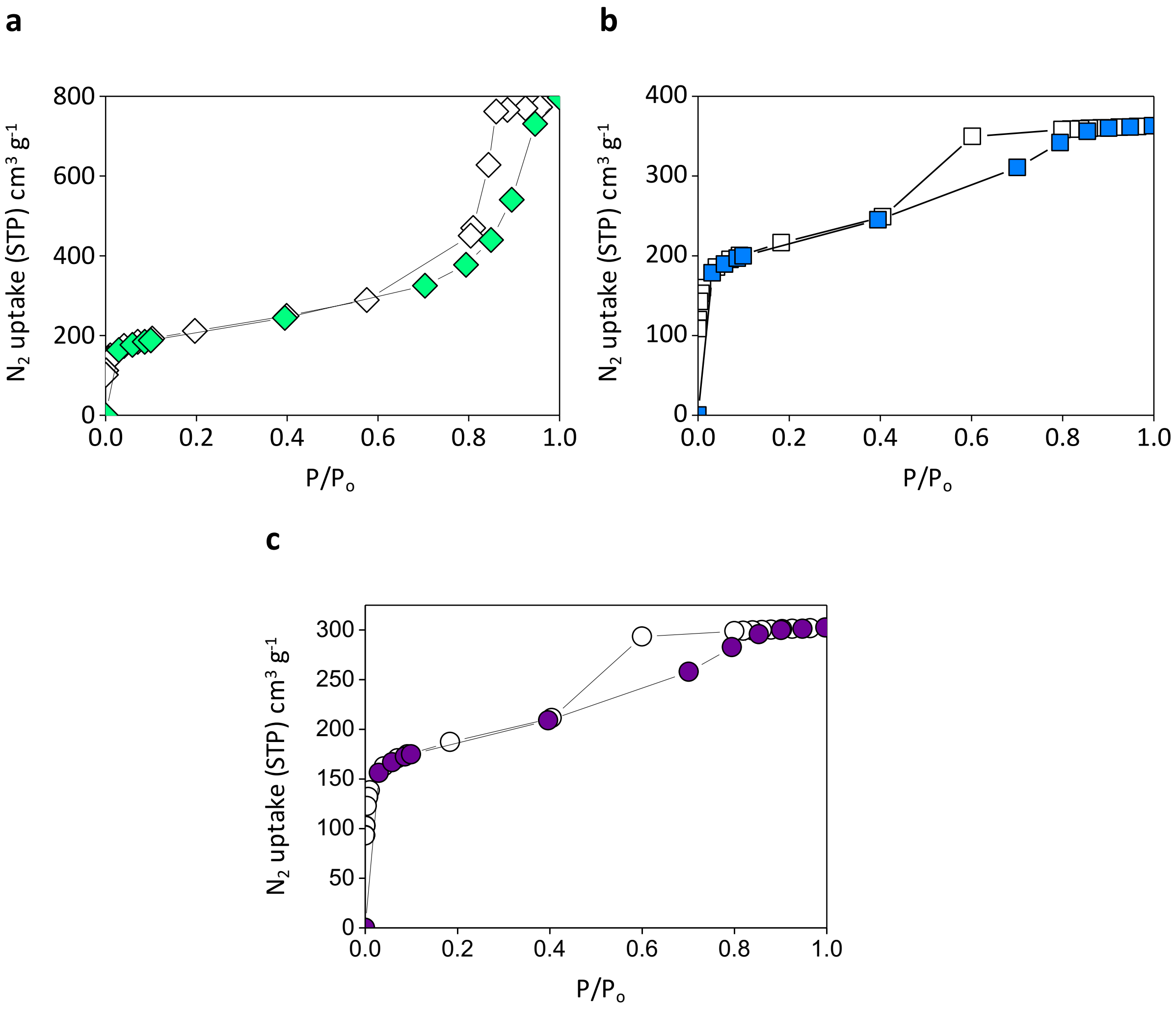

Supplementary Figure 34| N_2 adsorption-desorption isotherms for $_{mono}$UiO-66-NH_2. N_2 adsorption (filled marker) and desorption (hollow marker) isotherms (0-1 bar, 77 K) for UiO-66-NH_2 monoliths **a,** UiO-66-NH_2_A (green diamond), **b,** UiO-66-NH_2_B (blue square) and **c,** UiO-66-NH_2_C (purple circle) demonstrating hysteresis in both the micro- and mesoporous associated pressure ranges across all samples.

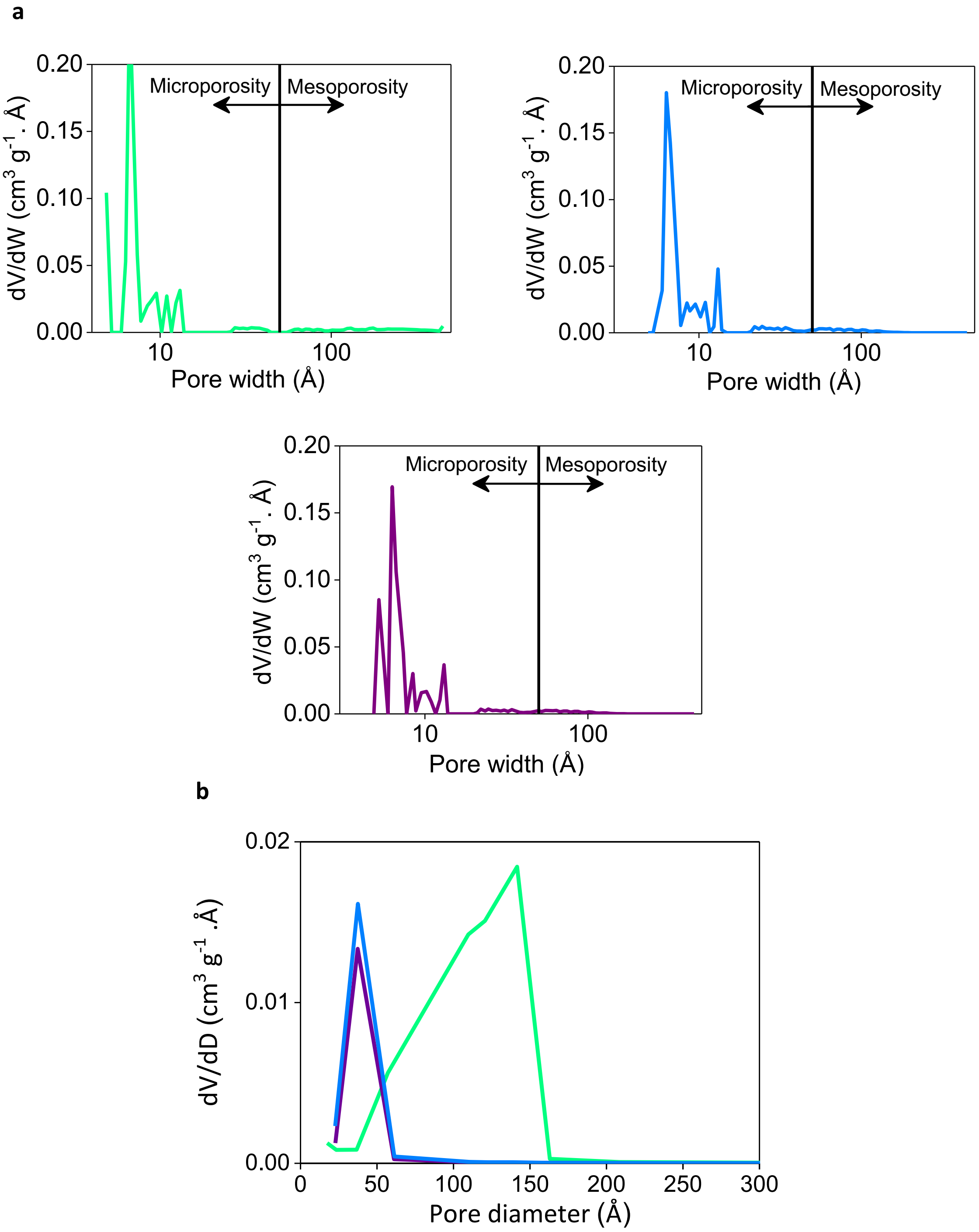

Supplementary Figure 35| Pore size distributions of $_{mono}$UiO-66-NH_2. a, Distribution of micro- and mesopore width and **b,** distribution of mesopore diameter across monolith samples: UiO-66-NH_2_A (green), UiO-66-NH_2_B (blue) and UiO-66-NH_2_C (purple), as obtained from Tarazona Non-Local Density Functional Theory (NLDFT) and Barrett-Joyner-Halenda (BJH) model analysis of N_2 isotherm data (Supplementary Figs. 30-34) respectively.

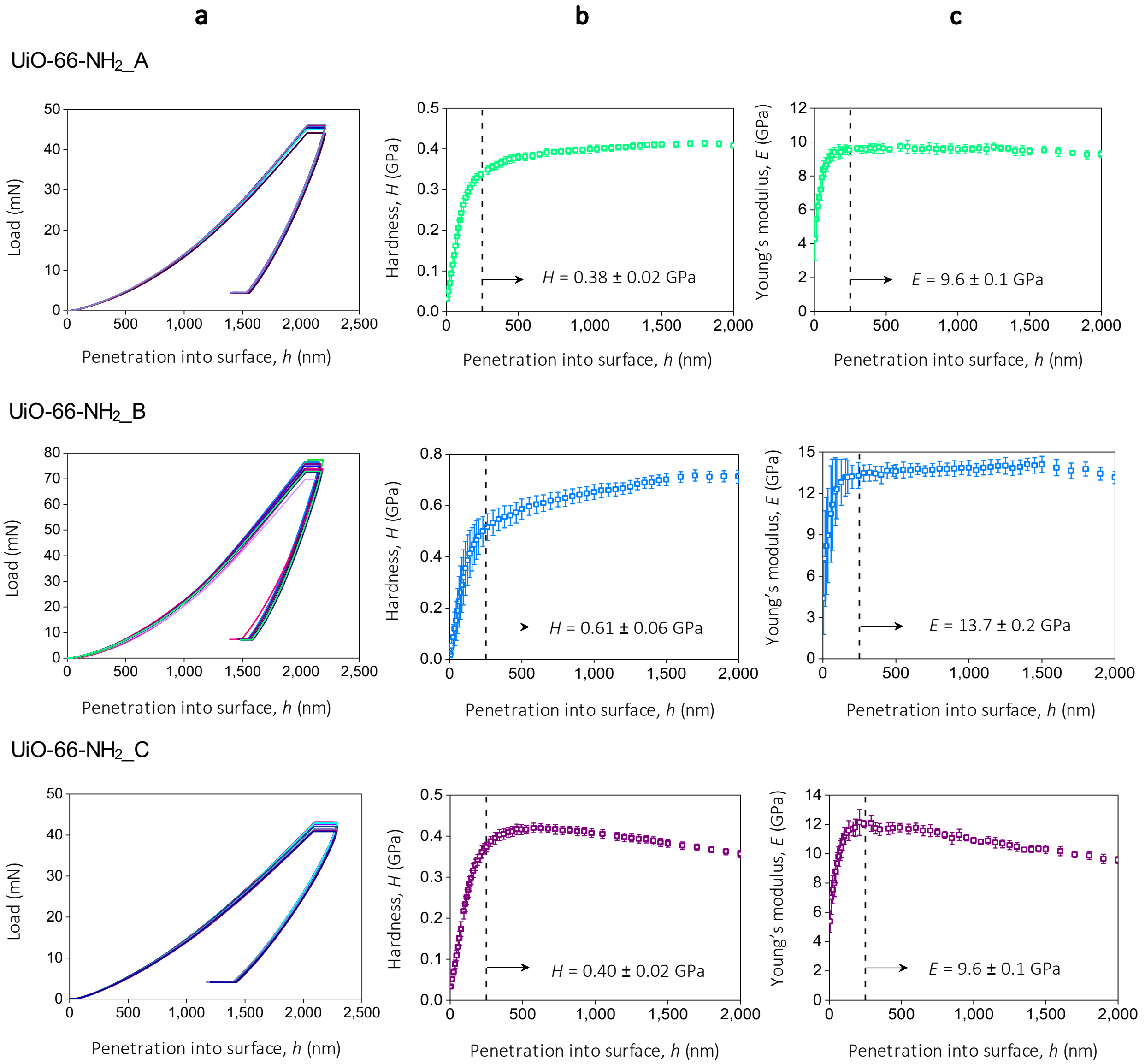

Supplementary Figure 36| Mechanical testing of $_{mono}$UiO-66-NH$_2$. Nanoindentation data for UiO-66-NH$_2$_A (green), UiO-66-NH$_2$_B (blue) and UiO-66-NH$_2$_C (purple) showing **a,** Load (mN) vs. Penetration into the monolith surface (*h*, nm) for 16 indents. **b,** The Hardness (*H*, GPa) and **c,** Young's modulus (*E*, GPa) is plotted as a function of Penetration depth into the monolith surface (*h*, nm). Mean properties and corresponding errors (inset) were obtained from measurements taken from 16 indents over penetration depths of 250-2000 nm, ensuring elimination of surface defects/tip artefacts.

Supplementary Methods 2| Synthesis of $_{mono}$NU-1000. Zirconium(IV) oxychloride octahydrate (121 mg, 0.375 mmol), 4-aminobenzoic acid (154 mg, 1.12 mmol) and trifluroacetic acid (200 µl) were dissolved in DMF (10 ml). 1,3,6,8-tetrakis(*p*-benzoate)pyrene (25 mg) was dissolved in DMF (10 ml). The solutions were combined at 140 °C and maintained at this temperature for 1 hour. The resulting yellow gel was collected by centrifugation (60 min, 5500 rpm) and washed according to Supplementary Table 6. The resulting $_{mono}$NU-1000 was activated under vacuum at 110 °C for 24 hours.

Supplementary Table 6| Experimental conditions for $_{mono}$NU-1000 synthesis

	Washing solvent	Centrifuge time	Drying temperature
$_{mono}$NU-1000	DMF (2 × 30 ml)<br>Acetone (2 × 30 ml)	4 × 60 min<br>1 × 60 min*	30 °C

*Additional 60 min centrifugation added after the last washing stage.

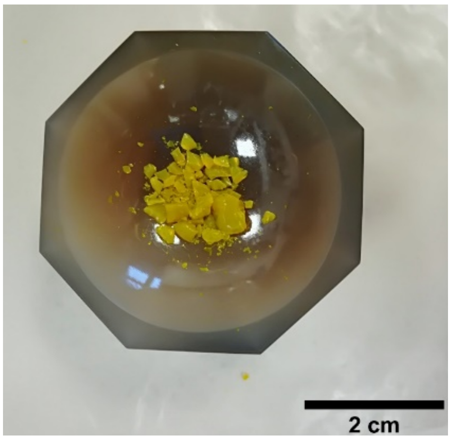

Supplementary Figure 37| Optical image of $_{mono}$NU-1000. Optical image of monolithic NU-1000. The sample was gently cracked with a pestle and mortar to show that when broken it does not crumble into powder but fractures, maintaining its monolithic form.

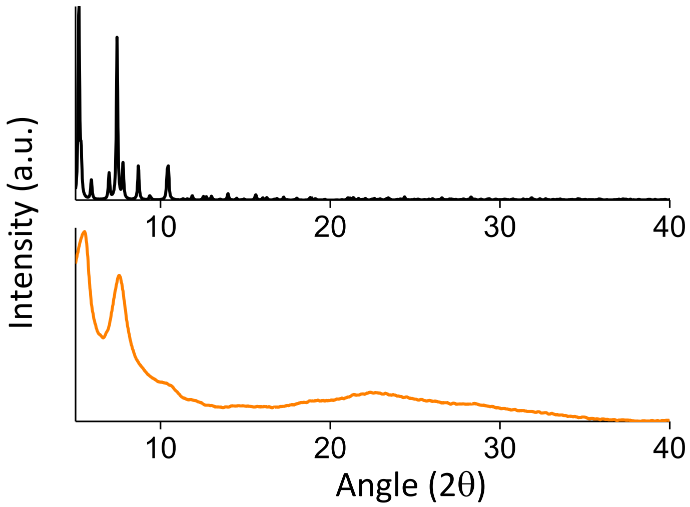

Supplementary Figure 38| X-ray diffraction of $_{mono}$NU-1000. Comparison of simulated XRD pattern of NU-1000 generated from its crystal structure; black,[24] to the experimentally obtained XRD pattern from $_{mono}$NU-1000 (orange). The observation of wide peaks in the latter XRD pattern is characteristic of line broadening due to the nano-crystalline MOF primary particles. While low angle major reflections (< 15 degrees) can be observed, the broad nature of the reflections masks lower intensity peaks at higher angles. This may further be linked to the presence of a secondary amorphous phase within the material – as observed by dark spots in the TEM images (Supplementary Fig. 39). This is further supported by the BET N_2 isotherm (Supplementary Fig. 41) which shows some degree of microporosity but low surface area compared to previous literature reports of the same material.[25] The large size of the NU-1000 linker (1,3,6,8-tetrakis-(*p*-benzoate)pyrene) reduces its solubility and makes highly microporous synthesis of the small nanoparticles needed for monolith creation difficult. These observations highlight the fact that, while a wide range of Zr-MOFs may be obtained as monoliths, the synthesis of highly crystalline materials using large/complex linkers may not be facile.

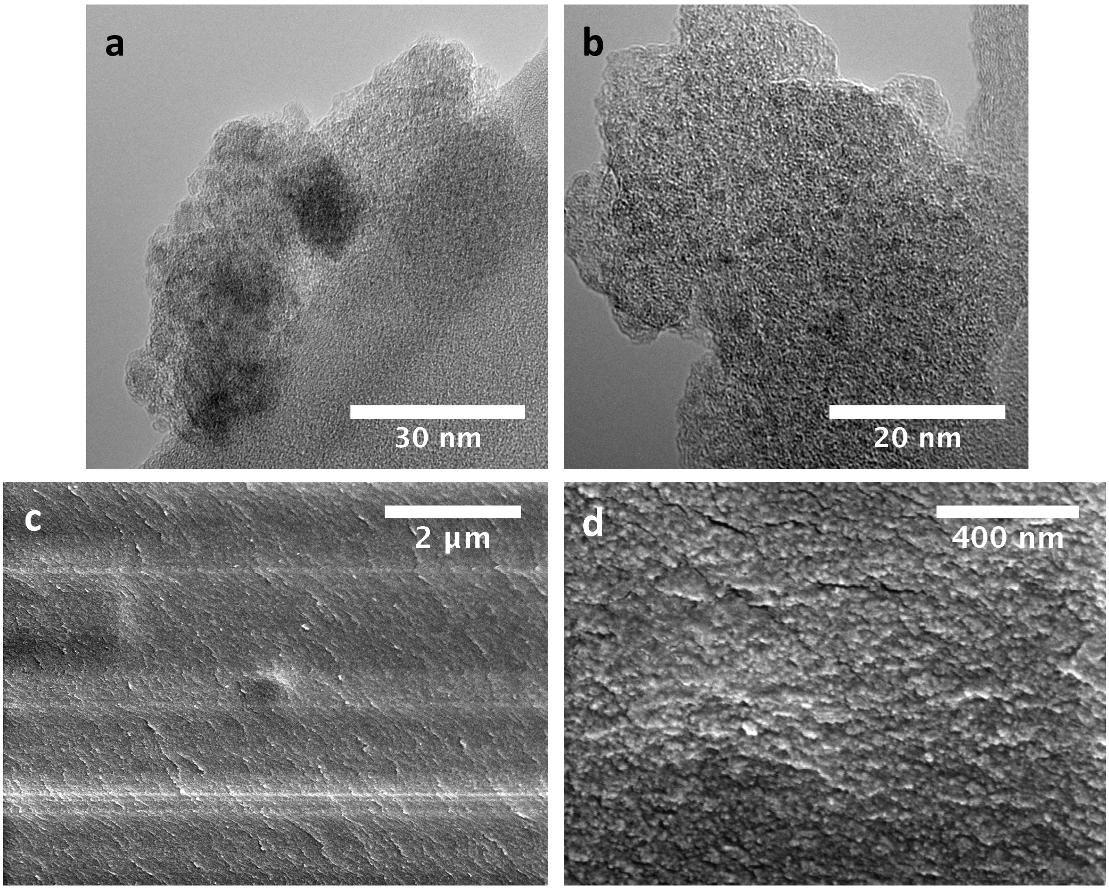

Supplementary Figure 39| Electron microscopy of $_{mono}$NU-1000. a, b, TEM images of $_{mono}$NU-1000 showing sub-10 nm primary MOF particles. These are comparable to TEM images of $_{mono}$UiO-66 and $_{mono}$UiO-66-NH_2. **b, c,** SEM images, low and high magnification respectively, of $_{mono}$NU-1000 showing the smooth surface of the monolith, which is resolved into a densely packed array of MOF nanoparticles upon increased image magnification.

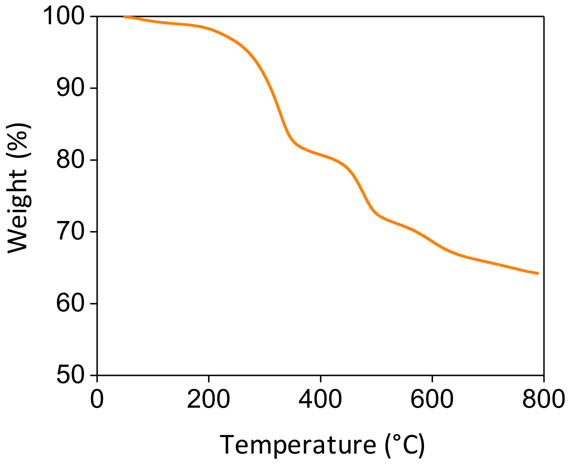

Supplementary Figure 40| Thermogravimetric analysis of $_{mono}$NU-1000. TGA analysis of $_{mono}$NU-1000 showing thermal decomposition over the temperature range 50-800 °C. The recorded graph shows a gradual decomposition before ca. 500 °C. This decomposition temperature is characteristic of highly stable zirconium MOFs which contain strong Zr-O bonds,[26] while the observation of gradual decomposition again supports the low crystallinity of the synthesised material.

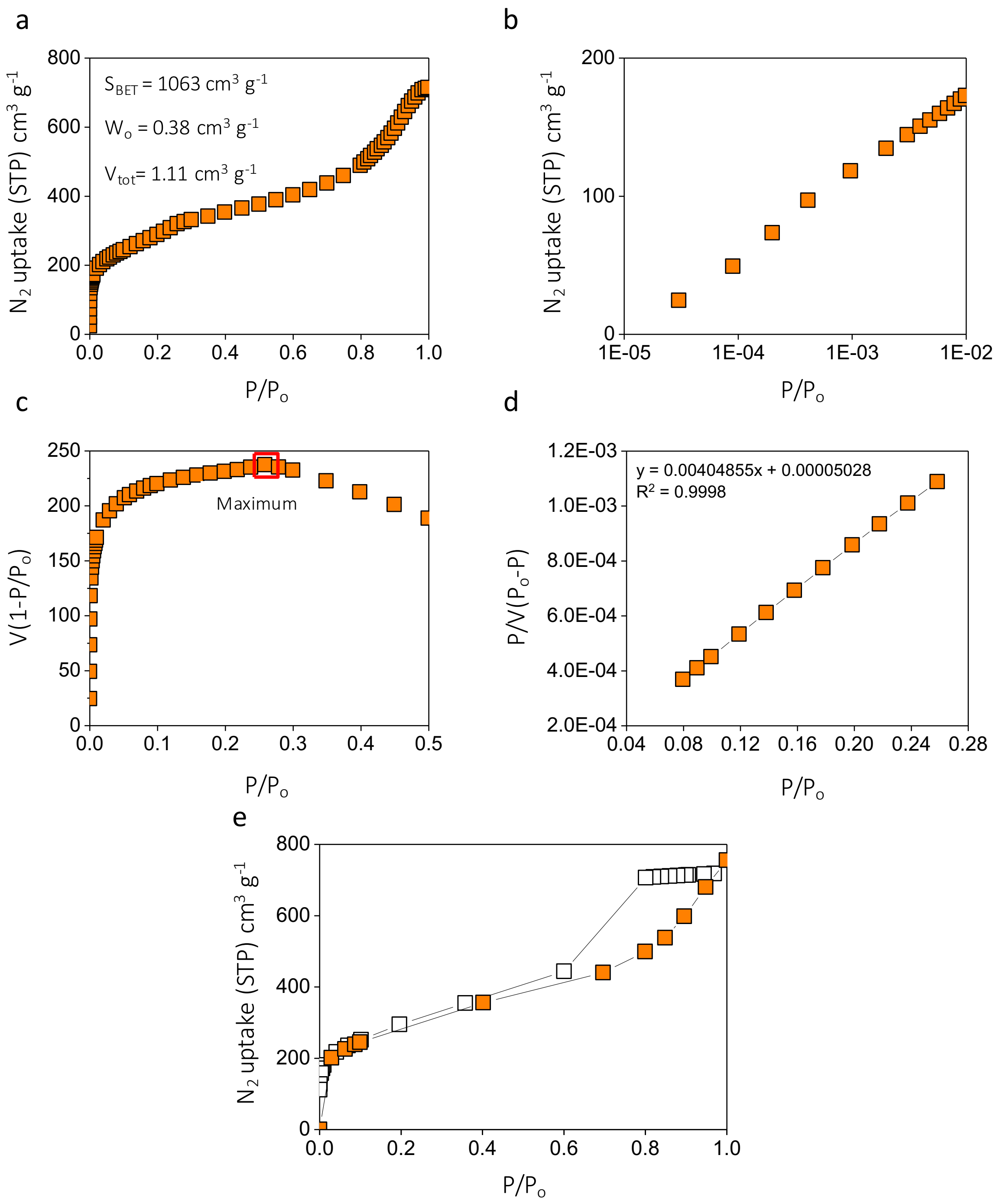

Supplementary Figure 41| N_2 isotherms and BET analysis of $_{mono}$NU-1000. **a, b,** Linear and semi-log plot respectively of N_2 adsorption isotherms at 77 K. **c,** Determination of the maximum P/P_o using Rouquerol's consistency criteria[10] and **d,** BET representation of N_2 isotherms showing the linear range utilised in surface area calculations for each monolith. The linear equation and R^2 value obtained from the linear fit to the data are given. **e,** Low pressure (0-1 bar) adsorption (filled marker) and desorption (hollow marker) isotherms demonstrating hysteresis during gas uptake and release in both the micro- and mesoporous associated pressure ranges. Data in **a,** BET area (S_{BET}), micropore volume (W_o) and total pore volume (V_{Tot}).

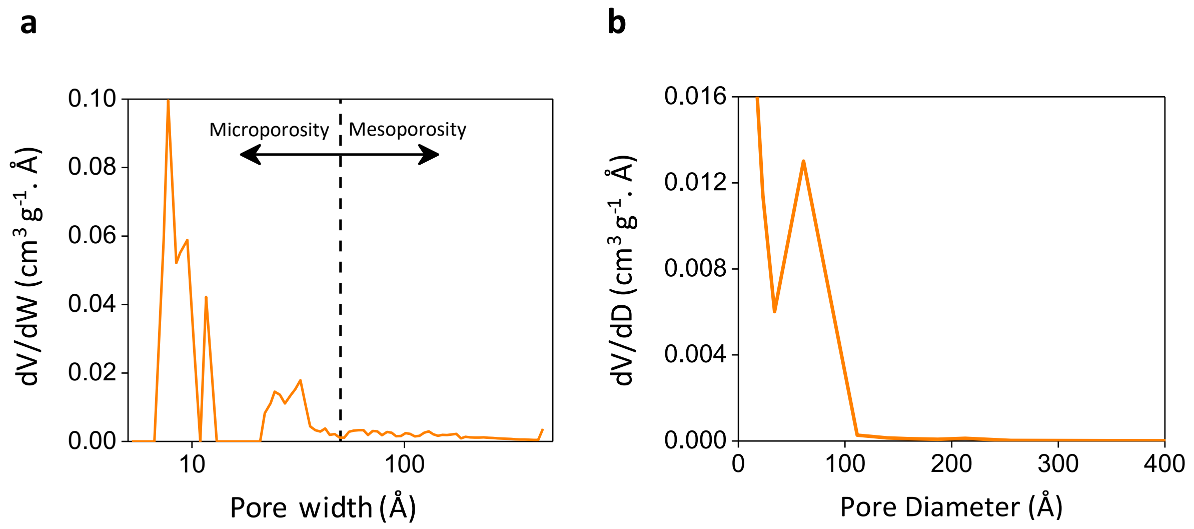

Supplementary Figure 42| Pore size distributions for $_{mono}$NU-1000. a, Distribution of micro- and mesopore width and **b,** distribution of mesopore diameter in $_{mono}$NU-1000 as obtained from Tarazona Non-Local Density Functional Theory (NLDFT) and Barrett-Joyner-Halenda (BJH) model analysis of N_2 isotherm data respectively.

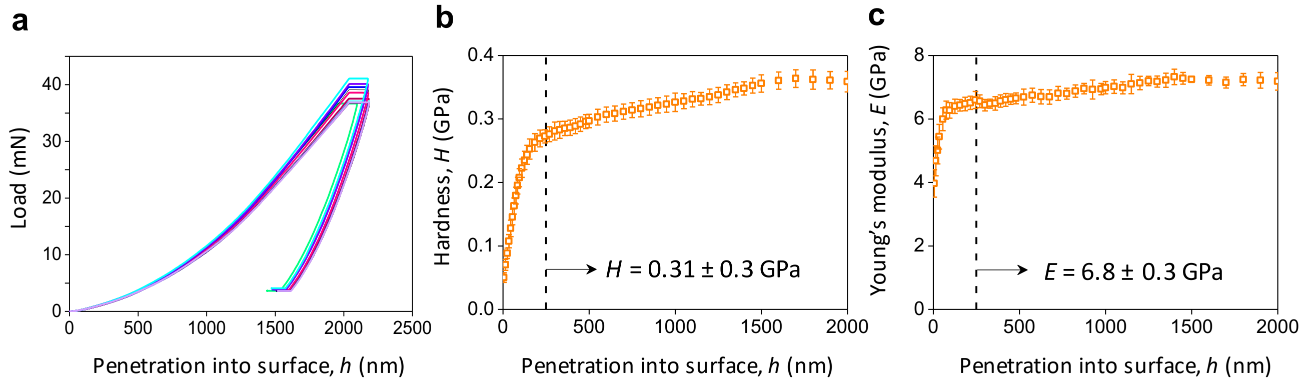

Supplementary Figure 43| Mechanical properties of $_{\text{mono}}$NU-1000. Nanoindentation data for $_{\text{mono}}$NU-1000 showing **a,** Load displacement raw data across 16 indents and **b, c,** Hardness (*H*, GPa) and Young's modulus (*E*, GPa) respectively as a function of Penetration depth into the monolith surface (*h*, nm). Mean properties and corresponding errors (inset) were obtained from measurements taken from 16 indents over penetration depths of 250-2000 nm, ensuring elimination of surface defects/tip artefacts.

Supplementary References

1. Bueken, B. et al. Gel-Based Morphological Design of Zirconium Metal-organic Frameworks. *Chem. Sci*. **8**, 3939–3948 (2017).

2. DeStefano, M. R., Islamoglu, T., Garibay, S. J., Hupp, J. T. & Farha, O. K. Room Temperature Synthesis of UiO-66 and the Thermal Modulation of Densities of Defect Sites. *Chem. Mater*. **29**, 1357–1361 (2017).

3. Orellana-Tavra, C. et al. Amorphous metal–organic frameworks for drug delivery. *Chem. Commun*. **51**, 13878–13881 (2015).

4. Wu, H., Yildirim, T. & Zhou, W. Exceptional mechanical stability of highly porous zirconium metal-organic framework UiO-66 and its important implications. *J. Phys. Chem. Lett.* **4**, 925–930 (2013).

5. Burtch, N. C., Heinen, J., Bennett, T. D., Dubbeldam, D. & Allendorf, M. D. Mechanical Properties in Metal-Organic Frameworks: Emerging Opportunities and Challenges for Device Functionality and Technological Applications. *Adv. Mater*. **1704124**, 1–18 (2017).

6. Thornton, A. W., Babarao, R., Jain, A., Trousselet, F. & Coudert, F.-X. Defects in metal–organic frameworks: a compromise between adsorption and stability? *Dalton Trans*. **45**, 4352–4359 (2016).

7. Tian, T., Velazquez-Garcia, J., Bennett, T. D. & Fairen-Jimenez, D. Mechanically and chemically robust ZIF-8 monoliths with high volumetric adsorption capacity. *J. Mater. Chem. A* **3**, 2999–3005 (2015).

8. Tian, T. et al. A sol–gel monolithic metal–organic framework with enhanced methane uptake. *Nat. Mater*. **17**, 174–179 (2017).

9. Digman, M. A., Caiolfa, V. R., Zamai, M. & Gratton, E. The phasor approach to fluorescence lifetime imaging analysis. *Biophys. J.* **94**, 14–16 (2008).

10. Gómez-Gualdrón, D. A., Moghadam, P. Z., Hupp, J. T., Farha, O. K. & Snurr, R. Q. Application of Consistency Criteria to Calculate BET Areas of Micro- and Mesoporous Metal-Organic Frameworks. *J. Am. Chem. Soc.* **138**, 215–224 (2016).

11. Dubbeldam, D., Calero, S., Ellis, D. E. & Snurr, R. Q. RASPA: Molecular simulation software for adsorption and diffusion in flexible nanoporous materials. *Mol. Simul.* **42**, 81–101 (2016).

12. Rappé, A. K., Casewit, C. J., Colwell, K. S., Goddard, W. A. & Skiff, W. M. UFF, A Full Periodic Table Force Field for Molecular Mechanics and Molecular Dynamics Simulations. *J. Am. Chem. Soc.* **114**, 10024–10035 (1992).

13. Wilmer, C. E., Kim, K. C. & Snurr, R. Q. An extended charge equilibration method. *J. Phys. Chem. Lett*. **3**, 2506–2511 (2012).

14. Potoff, J. J. & Siepmann, J. I. Vapor–liquid equilibria of mixtures containing alkanes, carbon dioxide, and nitrogen. *AIChE J.* **47**, 1676–1682 (2001).

15. Martin, M. G. & Siepmann, J. I. Transferable Potentials for Phase Equilibria. 1. United-Atom Description of n-Alkanes. *J. Phys. Chem. B* **102**, 2569–2577 (1998).

16. Lemmon, E. W., McLinden, M. O. & Friend, D. G. 'Thermophysical Properties of Fluid Systems'. in NIST Chemistry WebBook, NIST Standard Reference Database Number 69 (National Institute of Standards and Technology, Gaithersburg MD, 20899, 2018). doi:10.18434/T4D303

17. Myers, A. L. & Prausnitz, J. M. Thermodynamics of mixed-gas adsorption. *AIChE J.* **11**, 121–127 (1965).

18. Walton, K. S. & Sholl, D. S. Predicting Multicomponent Adsorption: 50 Years of the Ideal Adsorbed Solution Theory. *AIChE J.* **61**, 2757–2762 (2015).

19. Chen, K. et al. Enhanced CO2/CH4 separation performance of mixed-matrix membranes through dispersion of sorption-selective MOF nanocrystals. *J. Membr. Sci.* **563**, 360–370 (2018).

20. Wu, H. et al. Unusual and highly tunable missing-linker defects in zirconium metal-organic

framework UiO-66 and their important effects on gas adsorption. *J. Am. Chem. Soc.* **135**, 10525–10532 (2013).

21. Shearer, G. C. et al. Tuned to perfection: Ironing out the defects in metal-organic framework UiO-66. *Chem. Mater.* **26**, 4068–4071 (2014).

22. Browe, M. A., Napolitano, A., DeCoste, J. B. & Peterson, G. W. Filtration of chlorine and hydrogen chloride gas by engineered UiO-66-NH2metal-organic framework. *J. Hazard. Mater.* **332**, 162–167 (2017).

23. Garibay, S. J. & Cohen, S. M. Isoreticular synthesis and modification of frameworks with the UiO-66 topology. *Chem. Commun.* **46**, 7700 (2010).

24. Islamoglu, T. et al. Revisiting the structural homogeneity of NU-1000, a Zr-based Metal-Organic Framework. *CrystEngComm* **20**, 5913–5918 (2018).

25. Wang, T. C. et al. Scalable synthesis and post-modification of a mesoporous metal-organic framework called NU-1000. *Nat. Protoc.* **11**, 149–162 (2016).

26. Howarth, A. J. et al. Chemical, thermal and mechanical stabilities of metal-organic frameworks. *Nat. Rev. Mater*. **1**, 1–15 (2016).
